# Widespread Recombination, Reassortment, and Transmission of Unbalanced Compound Viral Genotypes in Natural Arenavirus Infections

**DOI:** 10.1371/journal.ppat.1004900

**Published:** 2015-05-20

**Authors:** Mark D. Stenglein, Elliott R. Jacobson, Li-Wen Chang, Chris Sanders, Michelle G. Hawkins, David S-M. Guzman, Tracy Drazenovich, Freeland Dunker, Elizabeth K. Kamaka, Debbie Fisher, Drury R. Reavill, Linda F. Meola, Gregory Levens, Joseph L. DeRisi

**Affiliations:** 1 Department of Biochemistry and Biophysics, University of California San Francisco, San Francisco, California, United States of America; 2 Department of Small Animal Clinical Sciences, College of Veterinary Medicine, University of Florida, Gainesville, Florida, United States of America; 3 Wildwood Veterinary Hospitals, Portola Valley, California, United States of America; 4 Department of Medicine and Epidemiology, School of Veterinary Medicine, University of California Davis, Davis, California, United States of America; 5 California Academy of Sciences, San Francisco, California, United States of America; 6 Kamaka Exotic Animal Veterinary Services, Mountlake Terrace, Washington, United States of America; 7 Acacia Animal Hospital, Escondito, California, United States of America; 8 Zoo/Exotic Pathology Services, West Sacramento, California, United States of America; 9 Arkansas Livestock & Poultry Commission Veterinary Diagnostic Laboratory, Little Rock, Arkansas, United States of America; 10 Cincinnati Zoo, Cincinnati, Ohio, United States of America; 11 Howard Hughes Medical Institute, Chevy Chase, Maryland, United States of America; University of Michigan, UNITED STATES

## Abstract

Arenaviruses are one of the largest families of human hemorrhagic fever viruses and are known to infect both mammals and snakes. Arenaviruses package a large (L) and small (S) genome segment in their virions. For segmented RNA viruses like these, novel genotypes can be generated through mutation, recombination, and reassortment. Although it is believed that an ancient recombination event led to the emergence of a new lineage of mammalian arenaviruses, neither recombination nor reassortment has been definitively documented in natural arenavirus infections. Here, we used metagenomic sequencing to survey the viral diversity present in captive arenavirus-infected snakes. From 48 infected animals, we determined the complete or near complete sequence of 210 genome segments that grouped into 23 L and 11 S genotypes. The majority of snakes were multiply infected, with up to 4 distinct S and 11 distinct L segment genotypes in individual animals. This S/L imbalance was typical: in all cases intrahost L segment genotypes outnumbered S genotypes, and a particular S segment genotype dominated in individual animals and at a population level. We corroborated sequencing results by qRT-PCR and virus isolation, and isolates replicated as ensembles in culture. Numerous instances of recombination and reassortment were detected, including recombinant segments with unusual organizations featuring 2 intergenic regions and superfluous content, which were capable of stable replication and transmission despite their atypical structures. Overall, this represents intrahost diversity of an extent and form that goes well beyond what has been observed for arenaviruses or for viruses in general. This diversity can be plausibly attributed to the captive intermingling of sub-clinically infected wild-caught snakes. Thus, beyond providing a unique opportunity to study arenavirus evolution and adaptation, these findings allow the investigation of unintended anthropogenic impacts on viral ecology, diversity, and disease potential.

## Author Summary

The facility with which viruses evolve underlies many of the problems they cause. Virus evolution is the reason we need a new flu vaccine each year. It’s how HIV and other viruses develop drug resistance. And it enables viruses to occasionally jump from animals to humans and cause new diseases. It is therefore important to understand how and under what circumstances viruses evolve. The arenaviruses are a group of viruses that infect mammals and snakes. Mammalian arenaviruses normally infect rodents but they can also infect humans, and, when they do severe and sometimes fatal disease can result. In this study, we studied genetic diversity in arenaviruses infecting captive snakes. We discovered an astonishing amount of diversity. Most snakes are infected by more than one virus strain, and these strains are merging and shuffling their genes (they are undergoing recombination and reassortment). The extent to which this is happening is exceptional, and has likely been caused by the importation and mixing in captivity of infected snakes from the wild. This provides an excellent opportunity to study the processes of virus evolution and may be an example of human activity altering its course.

## Introduction

Several mechanisms generate viral genetic diversity [1–3]. All work by producing populations of viral genomes, a small fraction of which may exhibit an adaptive advantage in a new host species, different tissue, or in the face of drug or immune pressure. Replication of RNA viral genomes by error-prone polymerases results in relatively high mutation frequencies, and RNA viruses replicate as collections of closely related variant genotypes [2,4–7]. Viral genomes can shed content or acquire new loci from their hosts by horizontal gene transfer. In coinfected cells, recombination between different viral strains or species can produce chimeric progeny [8–11]. And in cells coinfected by segmented viruses, reassortment generates virions containing shuffled mixtures of segments from the parental genotypes [11,12].

Understanding basic mechanisms of viral adaptation is essential in order to better combat, prevent, and predict viral diseases. For example, the ability of pandemic influenza virus strains to efficiently replicate in and be transmitted between humans frequently results from *de novo* mutation and reassortment [13]. The continuous emergence of drug-resistant genotypes is a major hindrance to the effective treatment of human immunodeficiency virus-1 and other pathogens [14,15]. And, recombination between individually attenuated strains present in the oral poliovirus vaccine results in neuropathic progeny strains, and complicates eradication efforts [16].

Viruses in the family *Arenaviridae* have bi-segmented single-stranded RNA genomes with a characteristic organization and gene repertoire [17–21]. The larger genome segment (L) is about 7 kb in length and encodes the viral RNA-dependent RNA polymerase (L) and a small zinc-binding RING domain protein (Z). The smaller segment (S) is about half as long and encodes the glycoprotein precursor (GPC) and nucleoprotein (NP). On each segment, the two viral genes are in opposite coding orientations and are separated by an intergenic region (IGR) that is predicted to form stable hairpin structures.

Two major lineages of arenaviruses have been described: those that primarily infect rodents and those that infect snakes. The rodent arenaviruses (proposed genus *Mammarenavirus*) typically establish chronic mild infections in their natural hosts but can be transmitted to humans and other mammals [22]. Severe disease such as Lassa hemorrhagic fever can result from these zoonotic infections. The snake arenaviruses (proposed genus *Reptarenavirus*) were first identified in US cases of inclusion body disease, a progressive and sometimes fatal disease best described in members of the *Boidae* and *Pythonidae* families (boas and pythons) [23,24]. The identification and study of snake arenaviruses in captive snakes in Europe corroborated and extended this finding [25–27]. One major difference between the snake and mammalian arenaviruses is the provenance of their GPC genes, with the snake virus gene being more closely related to the glycoprotein gene of filoviruses and some avian retroviruses [23,28].

Several mechanisms of arenavirus evolution have been described [17,29–31]. Like all RNA viruses, arenavirus genome replication is relatively error prone, and arenaviruses replicate as collections of related variants *in vivo* [32]. Recombination is thought to have given rise to the ancestral S segment of a clade of the New World rodent arenaviruses [33–35]. Recombination and reassortment between co-infecting arenaviruses has been observed in the laboratory [36–38]. And, it has been suggested that arenaviruses detected in snakes in Europe might have undergone recombination [39,40]. However, arenavirus recombination and reassortment have not been confirmed in natural infections involving extant species.

In this study we document and investigate viral genetic complexity of an unanticipated extent and form in naturally infected captive snakes. We determined the complete or near complete sequences of 210 viral genome segments using metagenomic sequencing. Sequencing results were corroborated and extended by discriminating quantitative reverse transcriptase PCR (qRT-PCR) and by tissue culture isolation experiments. We detected widespread recombination and reassortment. We also observed an unbalanced accumulation of multiple distinct viral genotypes in individual infections. These findings provide an opportunity to study basic mechanisms of virus evolution and fitness through the identification of genetic determinants underpinning their action.

## Results

### Sample collection and whole genome sequencing

In order to further characterize the genetic diversity of the snake arenaviruses, we gathered 123 frozen case and control tissue samples from around the U.S.A. that were collected between 1997 and 2014 (**S1 Table**). We screened these samples for snake arenavirus RNA using qRT-PCR with degenerate primers targeting the glycoprotein gene. A total of 56 samples tested positive by qRT-PCR for viral RNA. Clinical data of varying detail was available for samples. Histopathology was available for 58 of the 123 samples and detection of viral RNA was well correlated with histopathology-based IBD diagnosis (**Table 1**). However, many infected snakes displayed no overt clinical signs (**S1 Table**). Thus, detection of viral RNA was correlated with detection of inclusions in tissue sections, but not with obvious clinical measures in a straightforward fashion, consistent with previous reports [23,25,26,41].

**Table 1:** Detection of viral RNA is correlated with histopathology-based IBD diagnosis.

	Snake arenavirus RNA+	Snake arenavirus RNA-
IBD+ by histopathology	36^a^	5^b^
IBD—by histopathology	1	16

(a) Data for the 58 samples with histopathology-based IBD diagnoses are shown.

(b) For 2 of these 5, inclusions were observed in the brain, but not in other tissues, and frozen brain tissues were not available for PCR or sequencing.

We performed metagenomic sequencing to determine complete viral genome sequences. Samples from 98 animals, including the PCR-positive samples, were sequenced. Samples from 48 of the arenavirus-positive snakes were sequenced to a depth sufficient for assembly of complete or near complete viral genome segment sequences. In many cases, the assemblies included predicted terminal sequences (**S1 Fig**, [23,42]). In total, just over 1 million reads contributed to the assembly of 210 genome segment sequences (148 L and 62 S sequences) totaling 1.24 Mb.

Genome segment assemblies were validated by re-mapping paired-end reads [43]. Assemblies were well supported, with 99-fold median coverage (**S1 Fig**). Coverage levels were generally lowest in the intergenic regions (IGR), likely as a result of the predicted hairpin-forming sequences in these regions (**S1 Fig**). In cases where coverage levels in IGRs fell below 2 reads, PCR was used to confirm assembly continuity.

**Fig 1:**
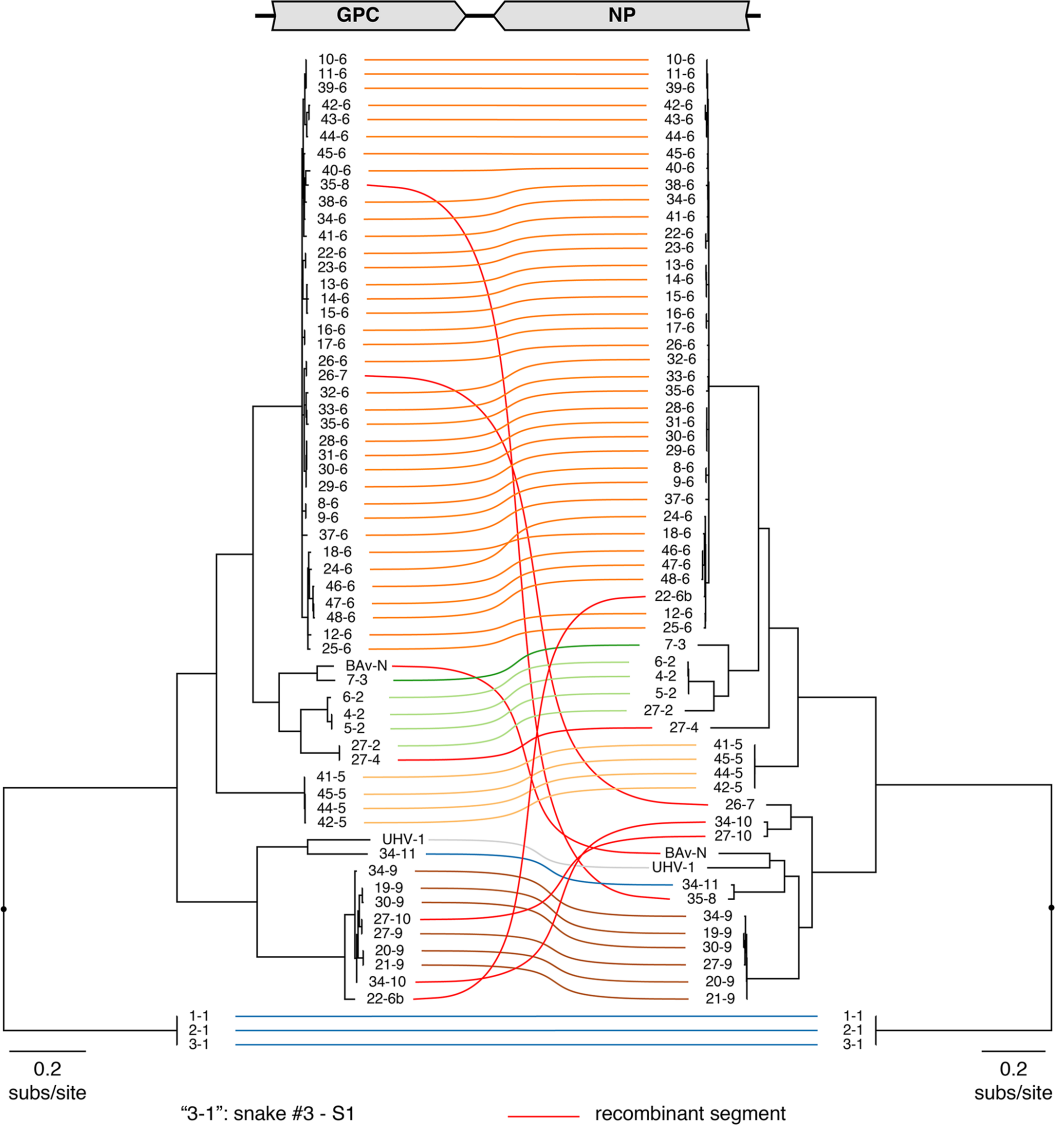
Numerous viral S segment genotypes were present in infected animals, including recombinant genotypes. Multiple sequence alignments of NP and GPC CDS were used to create a Bayesian phylogeny, which is depicted as a paired co-phylogeny. Lines connect CDS from individual genome segments. Lines are colored according to genotype. Red lines indicate recombinant genome segments listed in Table 2. Taxa are labeled by snake # and genotype #. For example “3–1” corresponds to snake #3 genotype S1. Sequences from snakes in Europe are labeled BAv_N (Boa AV NL) and UHV-1.

**Fig 2:**
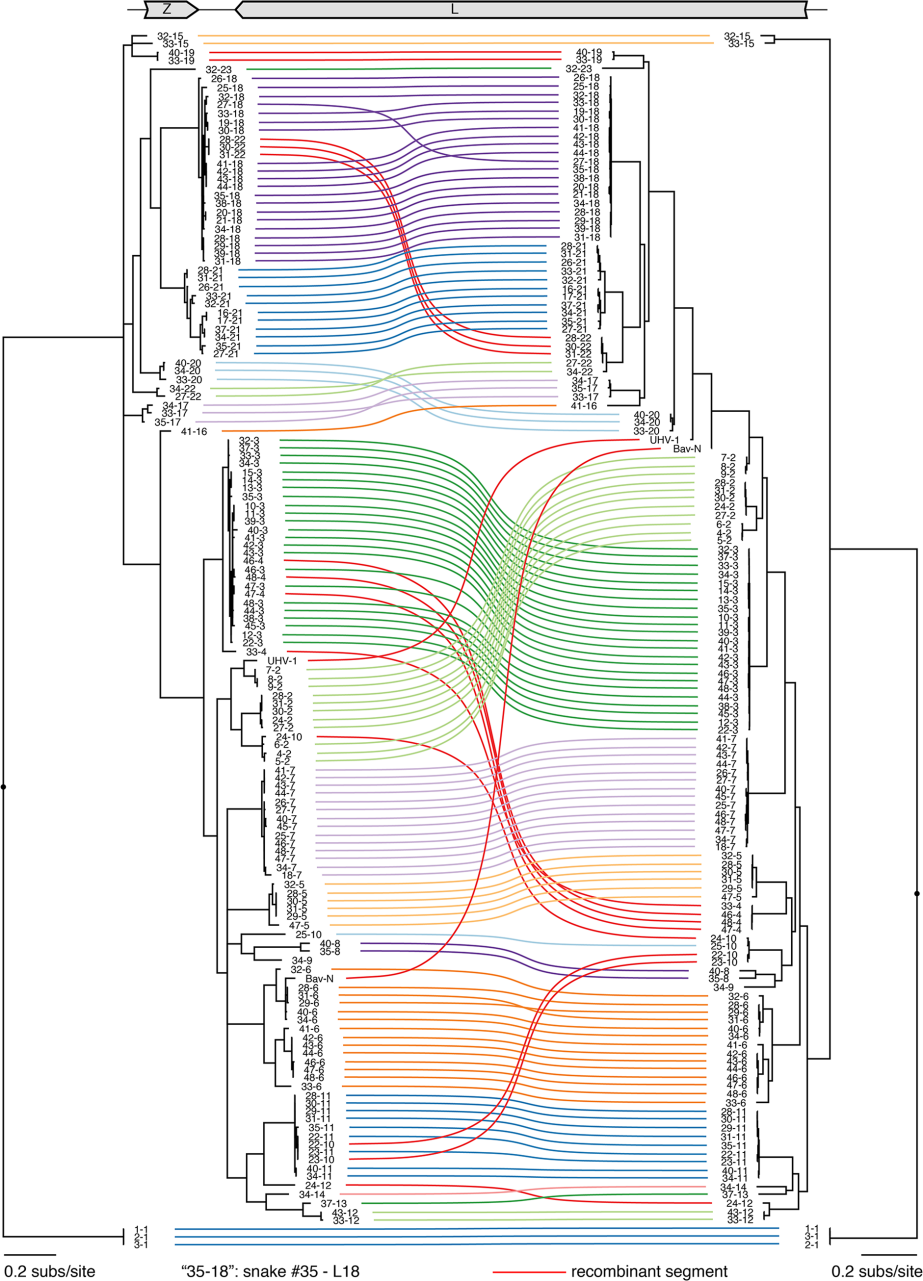
Numerous viral L segment genotypes were present in infected animals, including recombinant genotypes. Multiple sequence alignments of Z and L CDS were used to create a Bayesian phylogeny, which is depicted as a paired co-phylogeny. Lines connect CDS from individual genome segments. Lines are colored according to genotype. Red lines indicate recombinant genome segments listed in Table 2. Taxa are labeled by snake # and genotype #. For example “35–18” indicates snake #35 L18. Sequences from snakes in Europe are labeled BAv_N (Boa AV NL) and UHV-1.

**Fig 3:**
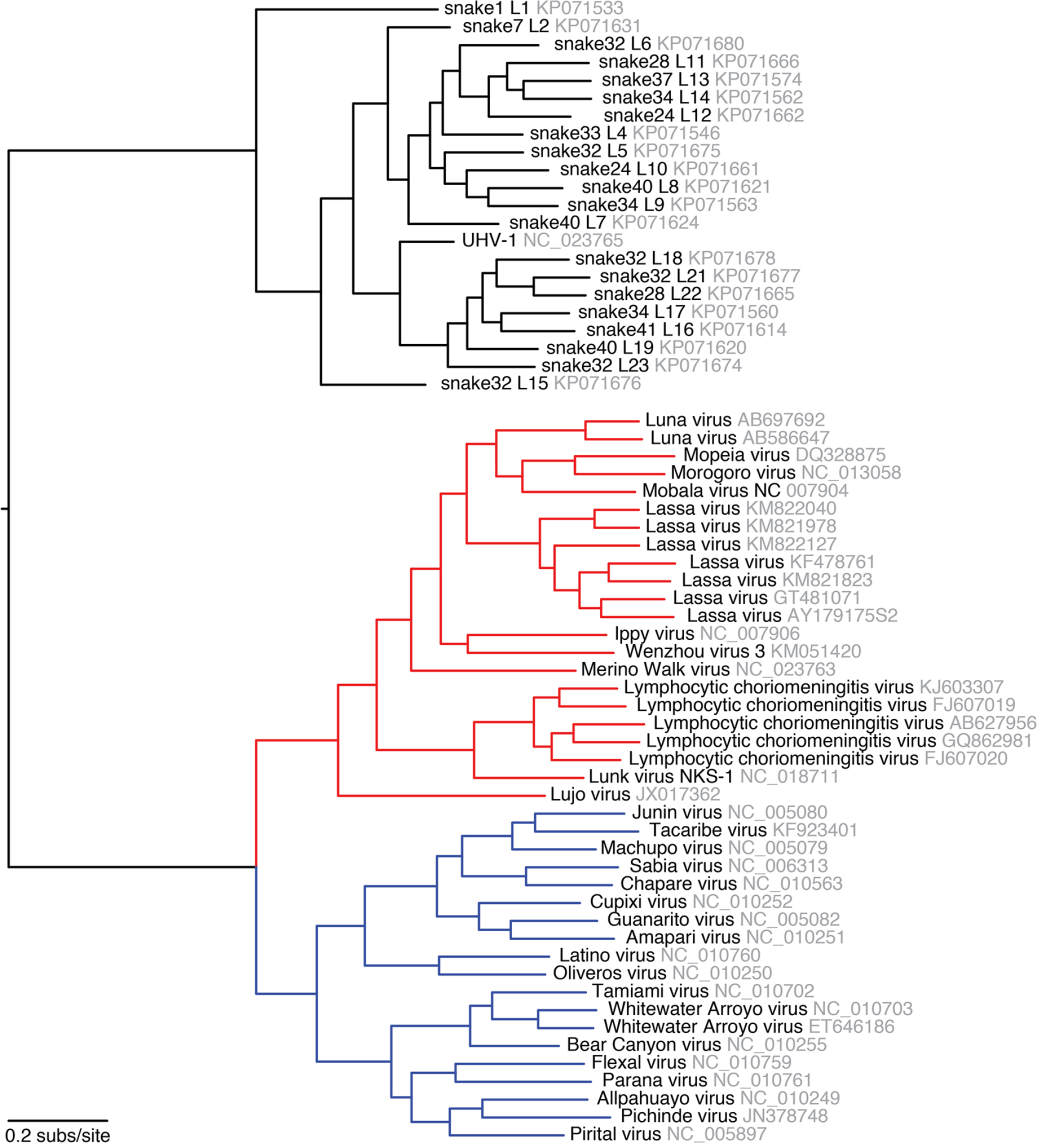
Phylogeny of representative snake and mammalian arenaviruses provides an overview of arenavirus diversity. Representative snake and mammalian arenavirus sequences were collected and used to create a multiple sequence alignment of L CDS, which was used to create a Bayesian phylogeny. Red lines indicate Old World mammalian arenaviruses and blue lines New World viruses. Sequences accession numbers are indicated.

Genome segments with less than 80% global pairwise nucleotide identity were classified into distinct genotypes (**Figs 1 and 2**and **S2 Fig**) [44]. A total of 11 S and 23 L genotypes were delineated by this criterion, and were designated S1-S11 and L1-L23. Within genotypes, L segment sequences shared a mean value of 96% pairwise nucleotide identity, and between genotypes, sequences shared 65% identity (**S2A Fig**). For S segments, these values were 96% and 64% (**S2B Fig**). Multiple alignments of the four coding sequences were used to create Bayesian phylogenies to visualize the inferred evolutionary relationship of these genotypes (**Figs 1–3**and **S3 Fig**).

**Fig 4:**
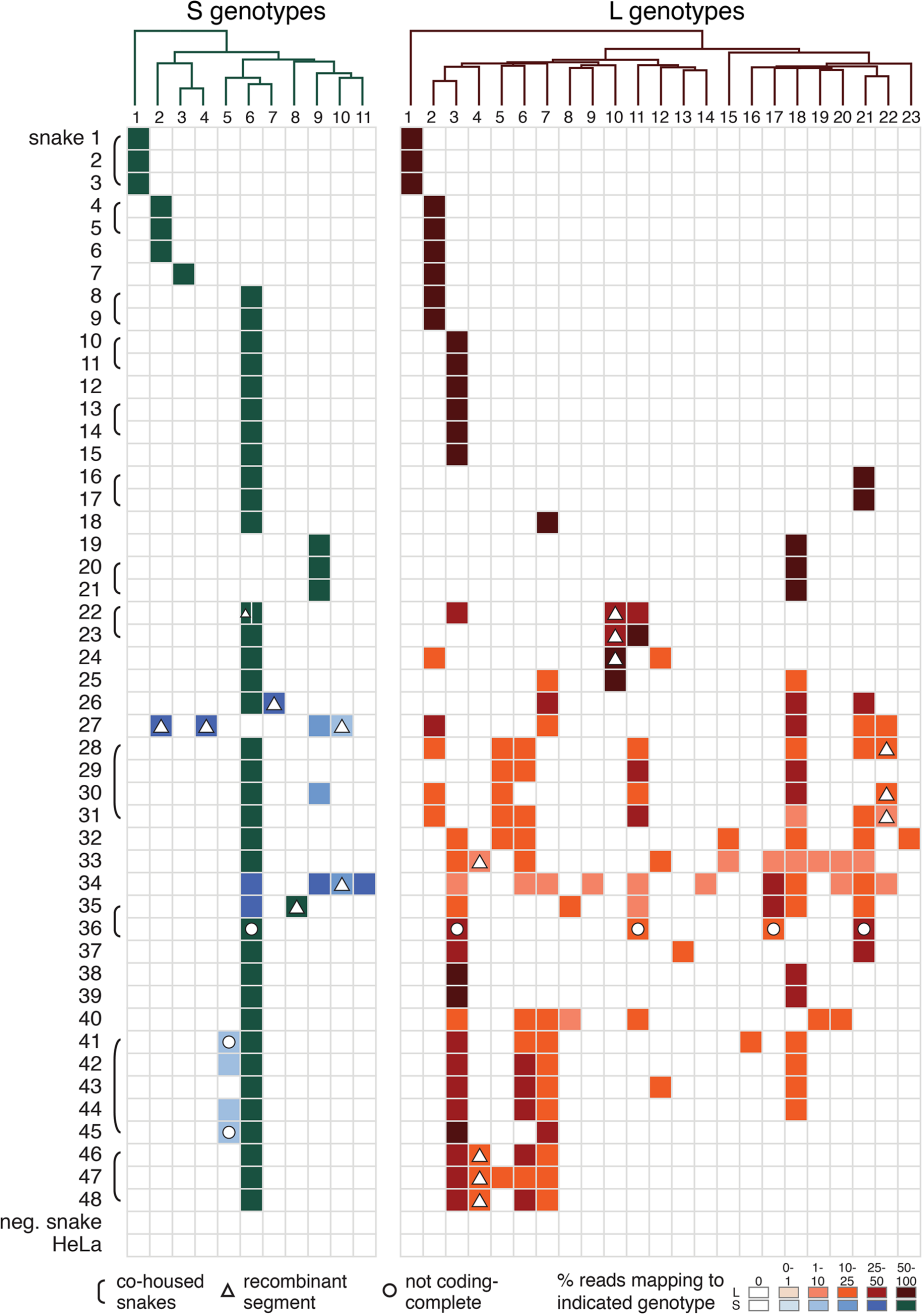
Individual snakes are infected by complex unbalanced sets of viral genotypes. These tables depict the fractional abundance of S and L genotypes detected in individual animals. Each row corresponds to an individual animal. Each column corresponds to a particular S or L segment genotype. Phylogenies on top of the tables were created using representative sequences from each genotype and a neighbor joining clustering method. Shading of cells indicates the fractional abundance of that genotype in the indicated animal, which was calculated as the proportion of sequencing reads mapping to that genotype divided by the total number of arenavirus-mapping reads from that animal. Recombinant segments are depicted with a triangle. All shaded boxes correspond to coding-complete assemblies, except for those indicated with a circle. Groups of animals harboring similar virus genotype combinations that were housed together are indicated with brackets. Neg snake is a sample from an uninfected snake and HeLa is a sample from total HeLa cell RNA.

In 21 of 48 infected snakes, viral genotypes consisted of a single S and a single L segment genotype (**Fig 4**and **S3 Table**, snakes #1–21). For example, snakes #1–3, the annulated tree boas from the California Academy of Sciences described in an earlier report, harbored S genotype 1 and L genotype 1. This S1/L1 genotype corresponds to the virus we referred to as “CAS virus” (CASV; [23]**)**. In snakes #4–6, segments of genotype S2 and L2 were detected, a genotype corresponding to “Golden Gate virus” [23].

Numerous reassortant genotypes were evident among the singly infected snakes (**Fig 4**). For example, the L2 segments present in snakes #7 and 8 were nearly identical at 98.6% pairwise identity, but their S segments shared only 73.5% pairwise identity (genotypes S3 and S6), indicating a more distant common ancestor. Similarly, the S6 segments in snakes #15 and 16 were 97.8% identical but the L3 and L21 segments in these snakes were only 62.2% identical. It was not possible in most cases to determine which S/L pairs were ancestral, nor when precisely reassortment had occurred, but it was clear that reassortment had produced these permuted pairings.

However, in the majority of animals (27 of 48 snakes), we observed viral genotypes that were substantially more complex than single S/L pairs. These snakes harbored multiple S and L genome segment sequences (**Fig 4**; snakes #22–48). The sets of viral genotypes ranged from that which would be expected for a straightforward co-infection by 2 virus strains in snake #45, to more complex combinations such as that in snake #33, which contained the sequences of 1 S and 10 distinct L segments. The animal with the most viral genotypes was snake #34, which contained 15 genotypes (4 S and 11 L) that could combine to produce virions with 44 unique S/L pairs (assuming coinfection of individual cells). The distinct L and S segment sequences within individual snakes shared on average only 65% and 70% pairwise nucleotide identity respectively, a level of variation not consistent with sequencing error or intrahost diversification. Snakes that were housed together often shared similar combinations of genotypes, and in these cases the sequences of these segments were closely related (**Figs 1 and 2**and **Fig 4**).

The accumulation of viral genotypes within individual animals was not balanced. In all cases there were more L than S segment genotypes (**Fig 4**and **S4 Fig**). On average, there were more than twice as many L segment genotypes as S genotypes per animal (mean values of 4.7 L and 2.4 S genotypes per multiply infected animal; **S4 Fig**). In fact, 18 of 27 animals with multiple L sequences harbored only a single S genotype.

The S6 genotype was dominant in individual animals and at a population level (**Fig 1 and Fig 4**). This genotype was first detected in a snake from Collierville, TN, and a partial S6 sequence was reported previously [23]. S segments of this genotype were detected in 37 of 48 snakes (77%). These sequences shared 96% average global pairwise nucleotide identity. Remarkably, the S6 segment genotype was found in combination with 21 of the 23 L segment genotypes described in this study.

**Fig 5:**
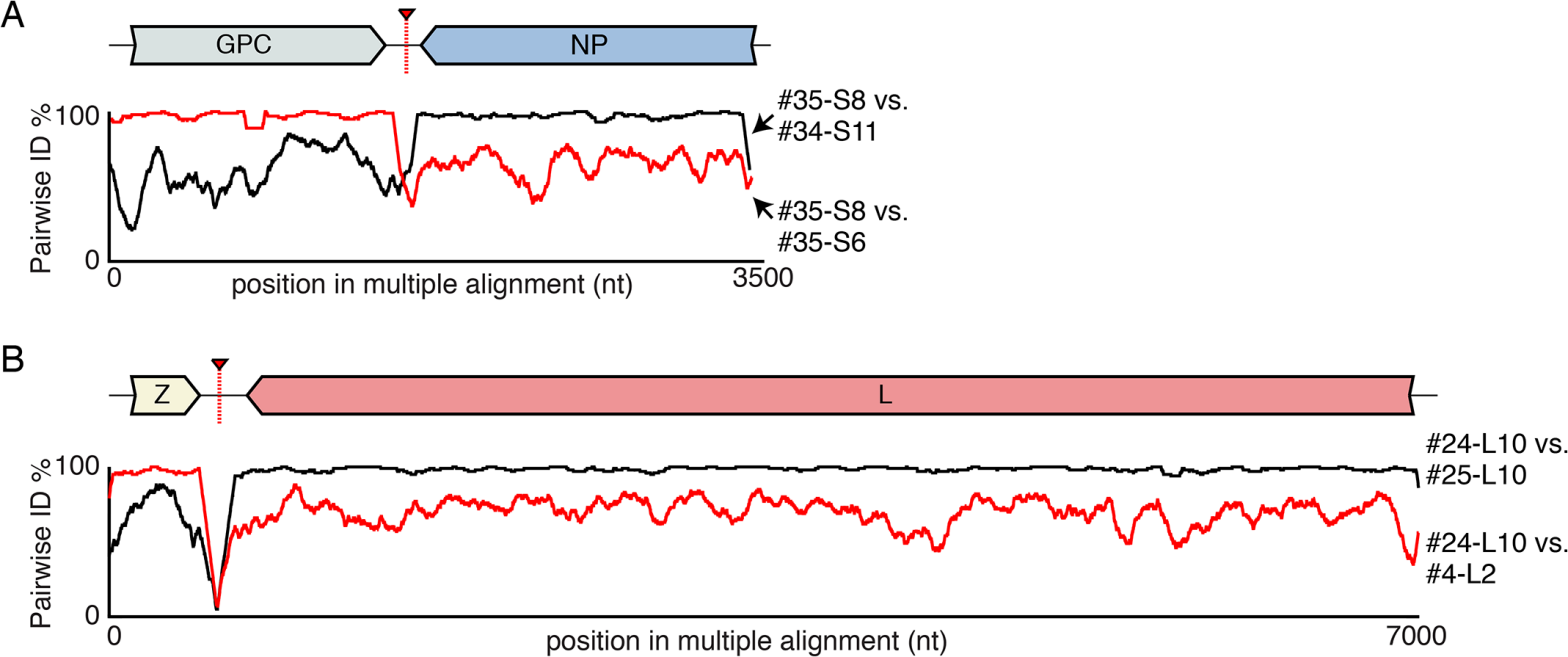
Examples of recombinant genotypes. **(A)** An example recombinant S segment: S8 from snake #35. Plots of pairwise nucleotide identity in 100 nt sliding window between this segment and two other 2 segments with sequences similar to presumed parental genotypes: S6 from snake #35 and S11 from snake #34. A cartoon of the recombinant segment is depicted, as is the approximate location of the recombination junction. **(B)** An example recombinant L segment (snake #24 L10) is depicted as in (A).

**Fig 6:**
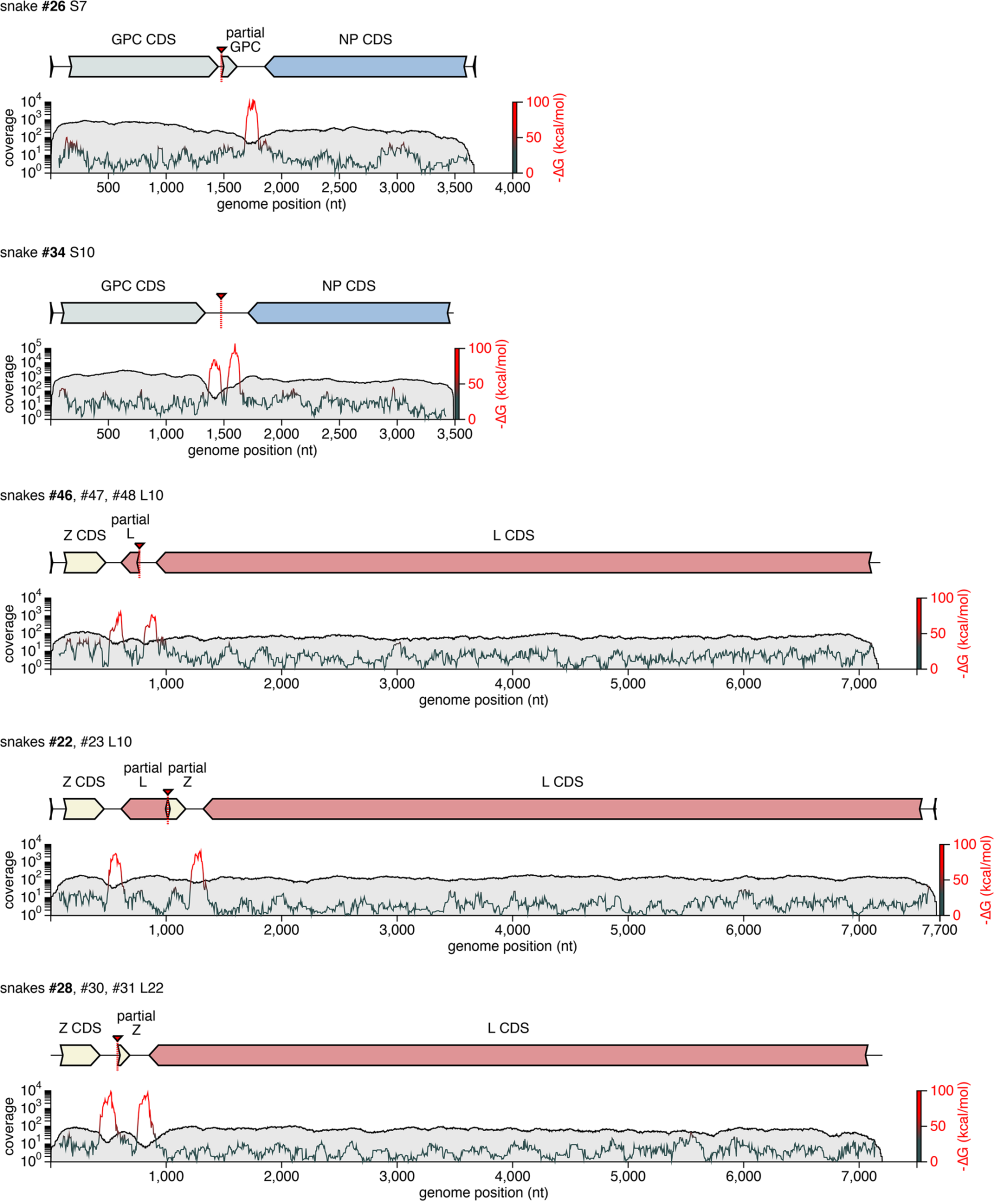
Recombinant genome segments with unusual organizations. Recombinant S and L genome segments with unusual double-intergenic regions (2xIGR) and/or partial coding sequences are depicted as cartoons. Plotted below each cartoon are coverage levels (the number of sequencing reads supporting each base in the assembly) and predicted free energy of folding (i.e. predicted RNA secondary structure; -∆G) of 140 nt sliding windows. Approximate locations of recombinant breakpoints are indicate with triangles and dotted lines. Partial coding sequences are indicated. Some of these segments, or very closely related versions thereof, were detected in multiple animals, as indicated. In these cases, cartoons and plots are based on the segment from the snake listed in bold font.

In addition to reassortant genotypes, we also identified 6 recombinant S segments and 8 recombinant L segments (**Figs 1 and 2**, **Figs 5 and 6, S1 Fig,** and **S2 Table**). We used the RDP4 software to detect and statistically evaluate support for recombination events (**Table 2;** [45]). Recombination events were well supported by RDP4 analysis and by read coverage levels over recombination junctions (**S1 Fig** and **Table 2)**. We also confirmed segment continuity by PCR amplification across junctions.

**Table 2:** Summary of recombinant genome segments identified.

**Recombinant S segments**			
**Snakes**	**Genotype**	**p-value** ^**a**^	**Notes**
#22	S6B	1x10^-164^	
#26	S7	6x10^-40^	
#27	S10	4x10^-57^	
#27	S4	4x10^-68^	
#34	S10	4x10^-57^	2x IGR^b^
#35	S8	3x10^-132^	
	Boa3 AV NL^c^	9x10^-13^	NC_023761.1
**Recombinant L segments**			
**Snakes**	**Genotype**	**p-value** ^**a**^	**Notes**
#22, 23	L10	2x10^-116^	2x IGR
#24	L10	2x10^-113^	
#24	L12	4x10^-63^	
#33, 40	L19	3x10^-9^	
#28, 30, 31	L22	1x10^-79^	2x IGR
#33	L4	2x10^-49^	
#41	L16	3x10^-38^	
#46, 47, 48	L4	2x10^-49^	2x IGR
	Boa3 AV NL^c^	2x10^-97^	NC_023762.1
	UHV-1^c^	1x10^-139^	NC_023765.1

**a)** Multiple comparison-corrected p-value reported by RDP4.

**b)** 2xIGR: segment contains 2 predicted hairpin-containing intergenic regions

**c)** Viruses isolated from European snakes in refs: [25,26]

While this analysis provided clear evidence of recombination, it was not always possible to determine which genotypes were parental and which were recombinant. However, it appeared that many of the recombinant segments coexisted in snakes with one of their parental genotypes. For example, in snake #35, 2 S segment sequences were evident. One of these was a canonical S6 genotype. The other segment, designated S8, shared 97% average pairwise nucleotide identity with the S6 segment’s GPC gene, but only 66% identity in the NP gene (**Fig 5A**). The S8 NP appeared to derive from a segment similar to the S11 NP found in snake #34. Similar patterns were observed for S segments in snakes #22, 26, 27, 34, and 35, and for L segments in snakes #22, 23, 28, 30, 31, 33, 46, 47, and 48 (**Figs 1 and 2**and **Fig 5B**). These may be situations where the parental and progeny genotypes persistently replicated together following a recombination event. Alternatively, the genotypes could have been acquired in independent transmission events.

Some recombination events resulted in unusual genome organizations. For example, the L10 genotype found in snakes #22 and #23 consisted of a full Z coding sequence and a partial L coding sequence from one parental segment concatenated to a partial Z and full L from a second segment (**Fig 6**). This segment was predicted to contain 2 intergenic hairpin-containing regions (2xIGR) separating the 2 L/Z pairs. Similar double-IGR segments and partial CDS were observed in recombinant S segments as well (**Fig 6**). An offset template switching event during genome replication may have generated these 2xIGR segments (**S5 Fig;** [10]).

We used several independent approaches to corroborate the original metagenomic sequencing. First, we completely re-sequenced 41 samples, using independent library preparations, and derived essentially identical results. Second, we developed a panel of PCR primer pairs that discriminated between distinct viral genotypes, and performed qRT-PCR on a subset of samples and genotypes. In all cases, qPCR-based genotyping mirrored sequencing results (**S6 Fig**). Third, we used tissue culture isolation as another means of determining viral genotype and to confirm that sequences corresponded to infectious virus (see below).

### Transmission of compound genotypes

**Fig 7:**
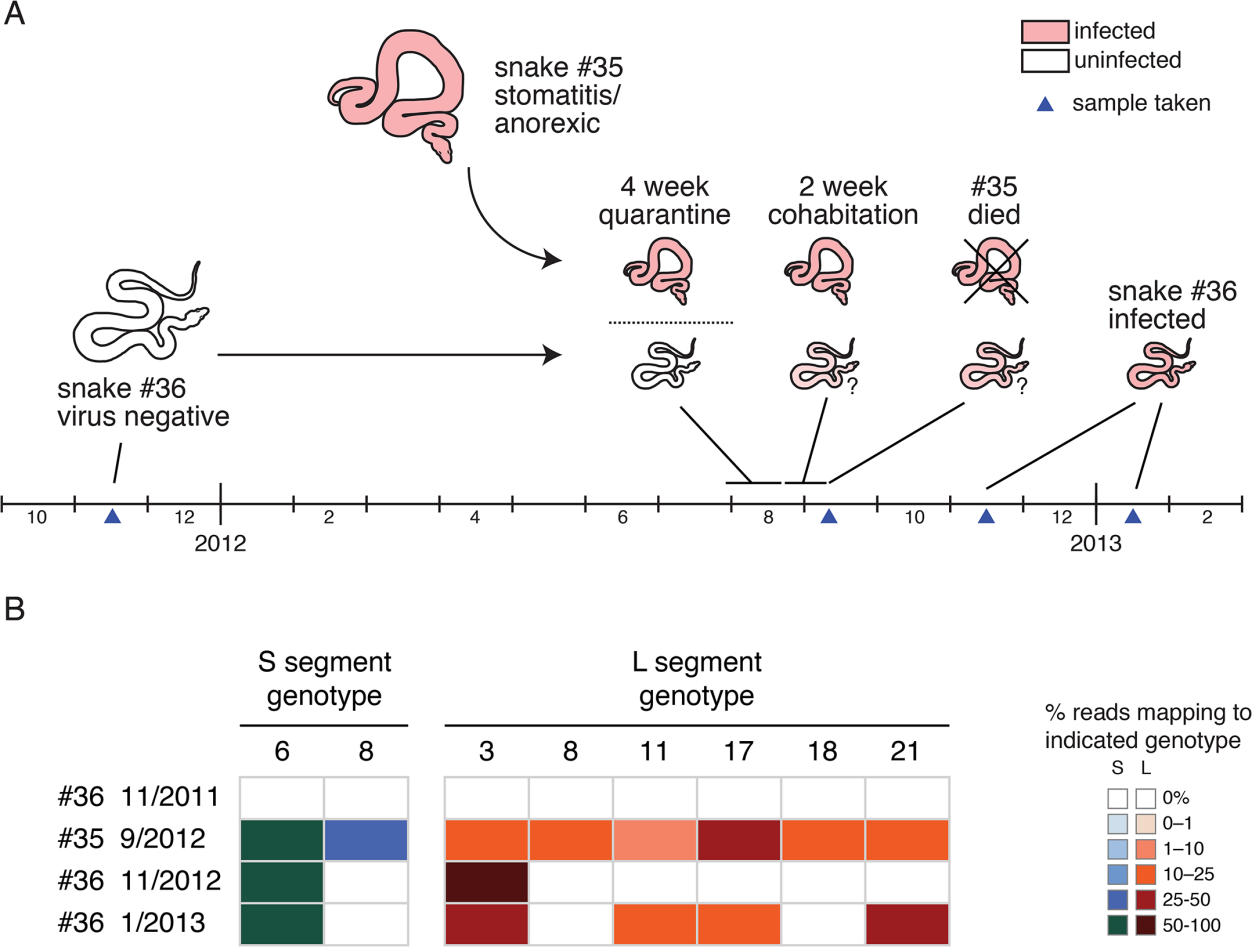
Transmission of multiple genotypes in a natural infection: (A) A cartoon and timeline depicting the circumstances surrounding the cohabitation of snakes #35 and #36. **(B)** Viral genotypes detected in snakes #35 and #36 at indicated time points. Fractional abundance of genotypes depicted as in Fig 4.

The introduction of an already infected snake (#35) into the proximity of an uninfected snake (#36) in a private collection enabled us to monitor viral transmission (**Fig 7**). A 2011 blood sample from snake #36 tested negative for snake arenavirus RNA by qRT-PCR and deep sequencing. Snake #36 was then not exposed to other snakes until September 2012, when its owners obtained a second snake, #35. Snake #35 arrived with stomatitis and was anorexic. Nevertheless, after a 4-week quarantine, snakes #35 and #36 were placed in the same enclosure. Snake #35 continued to refuse to feed and died two weeks later. We obtained the body of snake #35 and a blood sample from snake #36 taken in November 2012, and an additional blood sample from snake #36 from January 2013.

We determined that multiple genotypes were transmitted from snake #35 to snake #36 during their cohabitation (**Fig 7**). Snake #35’s viral genotype consisted of 2 S and 6 L genotypes (S6,8 / L3,8,11,17,18,21; **Fig 7B**). The November 2012 snake #36 blood sample was arenavirus positive, but the only genotypes detected by sequencing were S6 and L3. The January 2013 snake #36 sample was still positive, but now L genotypes 11, 17, and 21 were also detectable in the blood. Analysis of the viral sequences recovered from the two snakes revealed that they were closely related (98.5–100% identity). This data supports the transmissibility of compound unbalanced snake arenavirus genotypes in the context of cohabitation.

### Tissue culture isolation of unbalanced populations of viruses

**Fig 8:**
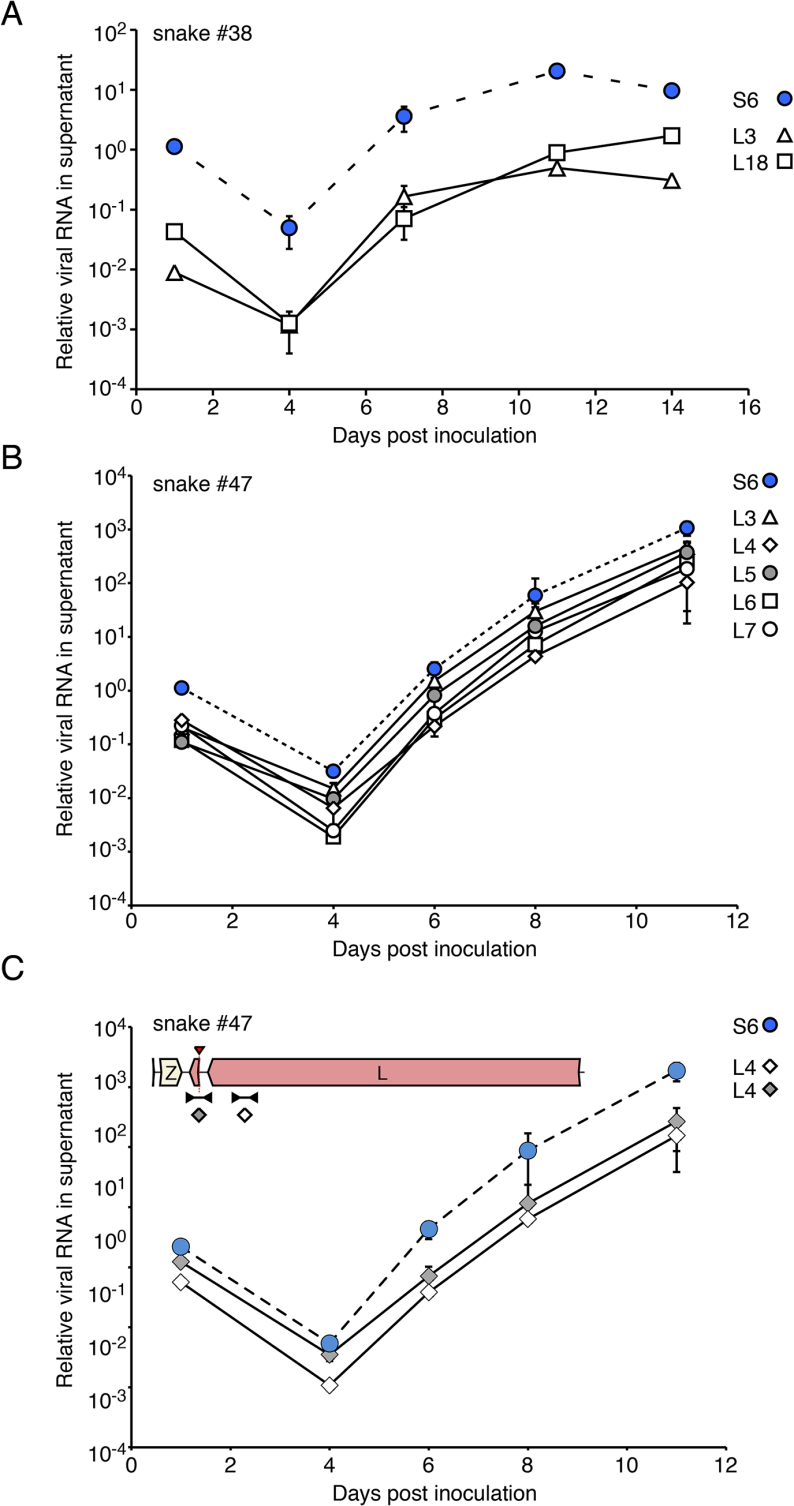
Virus populations replicate as stable ensembles in culture: (A) Liver homogenate from snake #38 was applied to cultures of JK cells and replication was monitored by measuring supernatant viral RNA levels using qRT-PCR and genotype-specific primers. Levels of distinct S and L genotypes detected are indicated and are normalized to the amount of S RNA detected in the first time point. Points and error bars represent mean and standard deviation of two independent experiments. **(B)** As in (A), but a liver homogenate from snake #47 was used as inoculum. **(C)** The 2xIGR L4 segment detected in snake #47 replicates stably in culture. Same experiment as (B), but qRT-PCR used primers that targeted two different regions of the L4 segment as depicted in the inset cartoon.

We performed tissue culture isolation and passaging experiments to investigate whether compound viral genotypes were competent to initiate productive infections. We applied homogenates from samples to cultures of boa constrictor-derived JK cells and monitored levels of virus RNA by qRT-PCR using genotype-discriminating primers. In all cases, we detected replication of all of the viral genotypes identified by our metagenomic sequencing (**Fig 8**). For example, snake #38 contained viral sequences of genotype S6/L3,18. When a liver homogenate from this snake was applied to a JK culture, replication of all 3 of these segments was detected (**Fig 8A**). Similarly, when a homogenate from snake #47 was used as inoculum, replication of all 6 expected viral genotypes was observed (S6 and L3,4,5,6,7; **Fig 8B**). The distinct L segments exhibited approximately equal replication efficiencies in these experiments, and the populations could be passaged to uninfected cell cultures. Thus, sequences of multiple viral genotypes corresponded to replication competent virus, and multiple viral genome segments replicated as stable ensembles in culture.

We also investigated whether an L segment with an unusual 2x IGR organization was competent for replication. Snake #47 L4 contains a partial Z region and 2 predicted IGR hairpins (**Fig 6**). To track this segment during infection, we performed qRT-PCR using 2 primer pairs: one pair that targeted the L gene of this segment and one pair that spanned the recombination junction (**Fig 8C**). Throughout the experiment, near equivalent amounts of template were detected using these 2 primer pairs, suggesting that most copies of this segment maintained their unusual structure.

**Fig 9:**
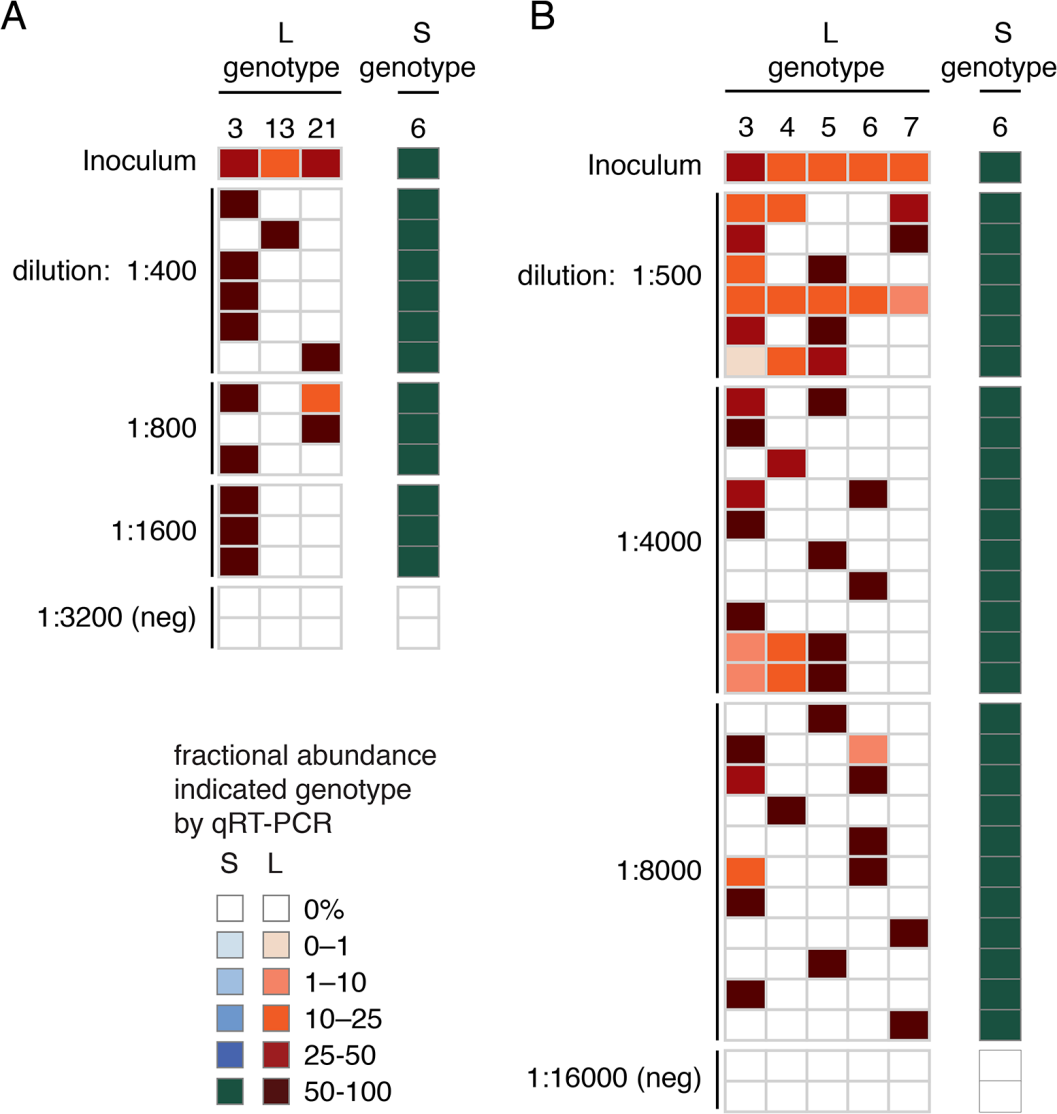
Most or all virus particles contain one L genotype. End-point dilution experiments were performed to determine the viral genotype of individual virus particles. Serial dilutions were prepared and applied to JK cells in 96-well plates. After 7–10 days, supernatant was reserved and wells were stained with anti-NP antibody to identify infected wells. qRT-PCR using discriminating primers was used to genotype the virus in individual well supernatant. Each row corresponds to one well and each column to an S or L genotype. The fractional abundance of S and L genotypes detected in individual wells are indicated as in Fig 2 as are the dilution used to inoculate the genotyped well. Negative wells (“neg”), not staining with anti-NP antibody, from the highest dilutions served as negative controls. The amount of each genotype detected in the inoculum is also indicated. Inoculum from snake #37 liver was used in (A) and from snake #47 liver in (B).

We performed endpoint dilution experiments to determine the genotypes of individual virus particles. We prepared dilution series from liver homogenates from snakes #37 and #47 and inoculated JK cells in 96 well plates. After 7–10 days, we transferred supernatants to new plates and stained cells with anti-NP Ab to determine wells positive for the presence of virus. Positive wells were then genotyped using discriminating qRT-PCR. In most cases, RNA from a single S and a single L genotype were detected in individual wells infected with the most dilute inocula (**Fig 9**). In 3 of 14 (21%) wells at these highest dilutions, more than one L genotype was detected. This could be the result of stochastic co-infection, clumped virus particles, or virus particles packaging more than one L segment. These results were consistent with the model that most virus particles packaged a single L segment, although we could not exclude the possibility that a minority of particles may package additional segments.

### Analysis of intrahost variation of individual genotypes

Intrahost variation for individual genotypes was also evident. For most genome segments, multiple sites with minor allele variants were detected (**S7 Fig**). The frequency of variants in most of these cases was less than 10 variant sites per kb (i.e., ≤1%; **S7 Fig**). In several cases, a higher frequency of variant sites was observed, for example the S6 segment of snake #41, which averaged 17 variant sites per kb (1.7% variants sites). This could have resulted from a greater degree of intrahost variation and divergence or from infection by viruses with closely related genotypes whose sequences were too similar to separate using short read assembly.

## Discussion

In this study, we surveyed the genetic diversity of arenaviruses infecting captive snakes in the USA. We used metagenomic sequencing and *de novo* assembly to determine genome sequences of viruses infecting 48 snakes. We found that most snakes were multiply infected by unbalanced ensembles of S and L segment genotypes. In total, we assembled 148 L and 62 S segment sequences that grouped into 23 L and 11 S genotypes. This expands the known diversity within this group of viruses by several fold. The high level of multiple infection has apparently given rise to numerous recombinant and reassortant genotypes, altogether compromising hundreds of unique viral combinations. We also discovered recombinant genotypes with non-canonical genome organizations, including those harboring apparently superfluous content. Metagenomic sequencing results were corroborated by PCR-based approaches, and extended by tissue culture isolation experiments. These findings highlight the utility of performing unbiased whole genome sequencing to determine pathogen genotypes. Indeed, our initial PCR-based screening correctly identified infected animals, but completely failed to uncover the genetic complexity present in the infections.

Although natural infection by multiple arenaviruses has not been previously documented, this phenomenon has been reported for other viruses. For example, infection involving up to 3 influenza viruses has been documented in humans and wild birds [46,47]. And, up to 7 or 9 distinct genotypes of torque teno virus or papillomavirus have been identified in individual human samples [48–50]. In plants, a virus isolate from citrus trees persistently infected by citrus tristeza virus was found to include several genotypes [51]. Shared characteristics of host-pathogen interaction may enable such highly multiple infections. These include persistent, sub-clinical viral replication, the absence of barriers to superinfection, the lack of an immune response capable of clearing infection, and a high prevalence of infection.

Although we detected many instances of snake tissues containing multiple viral genotypes, our results do not prove that individual cells in these animals were multiply infected. However, the detection of recombinant and reassortant genotypes suggests that at least in some cases cells are multiply infected.

Although multiple infection per se is not unprecedented, several aspects of these findings are. One is the apparent disconnect between the dynamics of the two viral genome segments, both in individual animals and at a population level. In individual animals, the accumulation of S and L segments was unbalanced: in all multiply infected animals, there were more L than S segments. In the most extreme case (snake #33), a single S genotype was paired with an ensemble of 10 L genotypes. It is possible that within animals the S and L segments inhabit different fitness landscapes. Another, not mutually exclusive, possibility is that differential replication kinetics or packaging efficiencies of the two segments may underlie the observed imbalance. Additional experiments in vitro and in animals will clarify this issue.

The population level dominance of the S6 genotype was also unexpected and is worthy of additional investigation. Genotype S6 segments were present in 37/48 infections (77%) and in 29 of these, no other S segment was detected. One possible explanation is that the S6 genotype replicates more efficiently within animals, or is more efficiently packaged and transmitted than other competing S segments. Alternatively, the high frequency of this genotype may be a stochastic effect, or may be proportional to viral genotypes in natural virus populations, from which these viruses in captive snakes presumably originate. Alternatively, it is possible that the 23 L genotypes observed here were originally paired with 23 S genotypes in free-ranging hosts. If this were the case, then 12 S genotypes are unaccounted for. Testing of wild-caught snakes could reveal the “missing” S genotypes and original S-L pairings and would reveal whether the S6 genotype has indeed risen to dominance in the context of captive animals.

Whether a similar degree of multiple infection is possible in mammalian arenaviruses is an open question, and one that may be relevant to the possible emergence of new mammalian arenavirus strains with pathogenic potential. It may be that there are larger species barriers for mammalian arenaviruses than there are for snake arenaviruses. Another possibility is that an ecological situation analogous to captive snake breeding has never been created for rodents. Alternatively, characteristics of the mammalian arenavirus host-pathogen interaction may prevent multiple infection. Indeed, superinfection exclusion has been documented in mammalian arenavirus tissue culture experiments [52–57]. And, cross-protection between mammalian arenaviruses has been documented *in vivo* [58–60]. Assuming that superinfection accounts for at least some of the genotype accumulation observed here, then no such mechanisms are operating in these snakes. Laboratory experiments with mammalian arenaviruses and other segmented viruses could test the generality of this phenomenon.

The discovery of “2xIGR” genome segment configurations was also unanticipated. It would be reasonable to predict that these segments would exhibit decreased fitness or be unstable during replication, given that they carry superfluous content. However, two lines of evidence suggested that these 2xIGR segments are capable of transmission and are stable over multiple rounds of replication. First, several of these segments were detected in co-housed snakes (L10 in snakes #22 and 23; L22 in #28, 30, and 31; L4 in #46–48). Presumably, each of these segments was initially generated via recombination in a single infected snake and then transmitted. Second, in tissue culture these segments replicated stably and could be passaged and isolated (**Figs 8C and 9B**). More extensive passage experiments in animals and culture will reveal whether maintenance of the 2xIGR configuration is disfavored over the long term.

These novel segment configurations also raise the possibility for the creation of payload-containing arenavirus genome segments. For example, the L10 segment found in snakes #22 and #23 contain 2 intergenic regions and 559 bases of extraneous incomplete coding sequence. If a suitable reverse genetic system were developed, this regions could be replaced with an internal ribosomal entry site and the 516 base NanoLuc luciferase gene or other payloads [61]. Such a tagged virus could be used for example in in vivo pathogenesis studies as an alternative to tri-segmented recombinant arenaviruses [62].

The nomenclature and taxonomy of the snake arenaviruses will likely have to be reconsidered in light of these findings [22]. We propose a nomenclature like that used for influenza A virus (IAV) subtypes, where new S and L genotypes are simply enumerated [63]. We would also propose following the taxonomic scheme for IAV subtypes, which belong to a single species, *Influenza A virus*. In this case, snake arenavirus genotypes could be grouped into one or possibly more species.

Recombinant genotypes were not limited to those described in this study. Discordance between GPC- and NP-based phylogenies including sequences from viruses detected in snakes in Europe suggested possible S-segment recombination [39,40]. The increased phylogenetic resolution enabled by this study confirmed the recombinant nature of the S and L segments of Boa3 AV NL and the L segment of UHV-1 [25,26] (**Table 2, Figs 1 and 2**).

It is possible that snake importation and husbandry practices have inadvertently created an ecological context that has enabled this phenomenon. Boa constrictors with different colorations (“color morphs”) are highly valued by collectors and breeders. Such colorations arise in nature as local adaptations and wild-caught snakes are commonly imported for breeding purposes. An estimated 98,500 boa constrictors per year were imported into the USA alone between 2005–2010 [64]. Mammalian arenavirus species have co-evolved with their distinct, geographically isolated rodent hosts, and it may be that snake arenavirus strains have co-evolved similarly in the wild. It is plausible that apparently healthy snakes persistently infected by various arenavirus species have been imported and intermingled in high-density breeding operations. This possible anthropogenic disruption of pathogen ecology is reminiscent of the influenza virus diversity generated in live animal markets [65]. Sampling of viral diversity in free-ranging snake populations are needed to clarify the impact of human activities on the evolution of these viruses and to further assess the disease potential of the resulting recombinant and reassortant genotypes.

In the absence of barriers to superinfection, an incalculable number of novel viral genotype configurations, made even more numerous by frequent intra-segment recombination and an error-prone polymerase, could rapidly evolve and accumulate within individual animals and in breeding facilities. In theory, this situation could be exacerbated by the introduction of mammalian arenavirus-infected rodents as feedstock [66], although it is unknown whether recombinant or reassortant mammalian/reptile arenaviruses are possible or viable. Regardless, further investigation of high-density reptile breeding and feed rodent facilities should be considered.

## Materials and Methods

### Sample collection

Samples were collected between 1997 and 2014 from California, Washington, Arkansas, Tennessee, Louisiana, Georgia, and Florida. Veterinarians in private practice or at university teaching hospitals collected samples. Samples were submitted to the University of Florida or the University of California San Francisco for further processing and storage. Blood samples were collected by cardiac or tail vein puncture and frozen until further processing. Tissue samples were collected during necropsy and frozen until further analysis or placed in formalin for histopathology. For histopathology, samples were preserved in 10% formalin, paraffin embedded, sectioned, and stained with hematoxylin and eosin. Board-certified pathologists blinded to infection status of samples examined H&E stained sections.

### qRT-PCR

RNA extracted from tissues (500 ng) or tissue culture supernatant was denatured for 5 min at 65°C then cooled on ice and added to 10 μl RT reactions containing 100 pmoles random hexamer oligonucleotide (MDS-286), 1× reaction buffer, 5 mM dithiothreitol, 1.25 mM (each) deoxynucleoside triphosphates (dNTPs), and 100 U SuperScript III (Life Technologies). Reaction mixtures were incubated for 5 min at 25°C then for 60 min at 42°C and then for 15 min at 70°C. cDNA was diluted 1:10 in 10mM Tris pH8, 0.1 mM EDTA. qPCR reactions contained 5 μl diluted cDNA, 0.5 μM each primer, 10 mM Tris pH 8.8, 50 mM KCl, 1.5 mM MgCl2, 0.2 mM each dNTP, 5% glycerol, 0.08% NP-40, 0.05% Tween-20, 0.15 μl Taq DNA polymerase, and 1x Sybr Green (Life Technologies) in each 15 μl reaction. Primer sequences are listed in **S2 Table**. Degenerate primers targeting the glycoprotein gene (MDS-400 and -402) were used to screen for infection. qRT-PCR to screen for individual S and L genotypes was performed using panels of primers that were designed to discriminate between the different sequences. Primer pair efficiencies were determined using template dilution series [67,68].

### Library preparation and sequencing

RNA was extracted from tissues as previously described [23]. Sequencing libraries were prepared as previously described [69]. Paired-end 2x135 bp sequencing was performed on an Illumina HiSeq 2500 in the Center for Advanced Technology at UCSF., producing an average of 2.0x10^6^ read pairs per sample.

### Sequence analysis

A stepwise pipeline was used to process sequencing data. First, data was demultiplexed. Then, 5 bases were trimmed of the 5′ end of reads and 1 base off the 3′ end. Next, low quality read pairs with any 10 base window with an average quality score below 30 were discarded. Then, reads sharing >98% global nucleotide identity (likely PCR duplicates) were collapsed using the cd-hit-est software version 4.6 [44]. Adapter sequences were trimmed from the ends of reads. Then, host-derived sequences were filtered as previously described [23]. Viral genome sequences were assembled from the remaining reads

An iterative strategy was used to assemble genome segment sequences. First, from each dataset, post-filtering reads were aligned using Bowtie2 to a database composed of all already-described snake arenavirus genome segments sequences [43]. Alignment parameters were set stringently (minimum alignment score of 150 in local mode alignment) so that only reads closely matching already described sequences aligned. Alignments were converted into BAM format using SAMTools software and inspected in Geneious software [70,71]. Sites differing from reference sequences were corrected to generate new draft genome sequences. Remaining virus-derived reads (determined by BLASTx, as described below) that didn’t align to an existing virus sequence were used to seed assemblies using the PRICE targeted de novo assembler [72]. PRICE contigs were added to the set of genome segment sequences and the process was reiterated until all reads were accounted for as described below.

Once a complete set of genome segment sequences was assembled, we used Bowtie2 to remap all reads from each dataset to the set of genome segment sequences derived from that dataset. These alignments were manually inspected and were used to generate coverage metrics. For each aligned base, coverage was only counted if the preceding and succeeding 3 bases also aligned. This “continuous coverage” metric is more conservative than a simple coverage metric and was implemented to identify possible incorrect assemblies. BAM files from these alignments, as well as FASTQ files of raw sequencing data for all snakes, have been deposited in the NCBI Short Read Archive (SRA; accession SRP057522). Genome segment sequences have been deposited in GenBank with accessions KP071471-KP071680.

We employed the following analysis strategy to confirm that we were accounting for the full viral genetic complexity in our datasets, i.e. that we were not overlooking any viral genome segments. We used the BLASTx tool to align translated reads from each dataset to a database containing all available snake arenavirus protein sequences, including the ones from this study. Because BLASTx alignments are based on protein sequence similarity, it is possible to use them to detect sequences with relatively distant homology. Thus, the number of reads with BLASTx alignments to arenavirus protein sequences (with E-value ≤ 10^–8^) determined a minimum expected number of virus sequences in each dataset. Then, we used the Bowtie2 aligner to stringently map reads from each dataset to the coding sequences of the assemblies generated from that dataset as described above. This allowed us to confirm that the assemblies accounted for all of the arenavirus-derived sequences in each dataset. To calculate the fractional abundance of individual genotypes, we divided the number of read pairs mapping to that genotype by the total number of arenavirus-mapping reads from that dataset.

### Phylogenetic analyses

We performed phylogenetic analyses to infer evolutionary relationships between viral genotypes. We created multiple sequence alignments of the coding sequences for each of the 4 viral gene coding sequences, using MAFFT version v7.017 with default parameters [73]. These alignments were trimmed using the Gblocks software version 0.91b using default parameters except allowing up to half gaps in columns (parameters:-t = d-b5 = h [74]). We used these trimmed alignments and the JModelTest software v2.1.6 to identify a best-fit nucleotide substitution model (GTR; [75,76]). We ran this software with parameters:-s 11-f-i-g 4-AIC-BIC-AICc-DT-p-a-w. We used MrBayes 3.2.2 to create Bayesian phylogenies from these alignments, using commands lset nst = 6 rates = propinv and mcmc ngen = 2000000 [77]. These phylogenies were visualized using FigTree software (http://tree.bio.ed.ac.uk/software/figtree/).

To create phylogenies including representative snake and mammalian arenavirus sequences, we first downloaded all sequences from the NCBI nucleotide database w/ query: “txid11617[Organism:exp]”, i.e. all sequences annotated as being of arenavirus origin. We removed sequences that were not complete or not coding-complete. We extracted NP and L CDS from these sequences. We used cd-hit-est to create a set of representative sequences, with sequences sharing >80% pairwise nucleotide identity collapsed (-c 0.8) [44]. We then created and trimmed multiple alignments and phylogenies as described above.

### Detection of recombinant genotypes

Global multiple sequence alignments of all S and all L segments were created using MAFFT software version 7.017 with default parameters [73]. Full genome sequences for described European snake arenavirus isolates were included in these alignments (University of Helsinki virus (UHV-1) and Boa arenavirus NL; NCBI accessions NC_023766.1, NC_023765.1, NC_023761.1, and NC_023762.1). Alignments were analyzed with the RDP4 recombination detection program version 4.39 using default parameters except to specify linear molecule topology [45]. We required that recombination events be detected by at least 2 of the methods implemented in the software. Putative recombinant segments were validated by examination of phylogenetic discordance and pairwise sequence alignments.

### RNA secondary structure analysis

Genome segments sequences were divided into 140 nt sliding windows (the approximate size of intergenic regions) offset by 5 nt. CentroidFold v0.0.9 was used to calculate the minimum free energy of folding for each window using parameters-g 4-e CONTRAfold [78].

### Analysis of variant sites

Variant sites were called using SAMTools version 0.1.19, using command mpileup—I. We required that variant sites be supported by at least 4 reads in the context of a minimum coverage level of 20 total reads. The number of variant sites per genome segment was calculated and normalized to the length of each segment.

### Tissue culture

RIC For inoculation experiments, frozen tissue samples were thawed on ice and homogenized in MEM + 25mM HEPES (SF-MEM) using a Dounce homogenizer. Homogenates were clarified by centrifugation at 10,000g for 2 minutes then passed through a 0.45 μm filter. Filtrates were diluted 1:10 in SF-MEM then added to cultures of near confluent JK cells. Culture medium was replaced periodically and supernatants were stored at -80°C until further analysis.

### Monitoring of viral replication in culture

We used qRT-PCR to measure viral RNA levels in culture supernatant. We extracted RNA from 180 μl supernatant using the Zymo viral RNA kit (Zymo Research). 5 μl RNA (25% of eluate) was used as template in RT reactions as above. Resulting cDNA was diluted and used in qPCR reactions as above, with primers listed in **S2 Table**. Primer pair efficiencies were calculated as above and used to determine quantities of viral RNA relative to the amount of S segment RNA present in the first time point sample.

### End-point dilution experiments

JK cells were grown as described above and were plated at a density of 5000 cells per well in 96-well plates. One day later diluted virus stocks were added to cells. Cells were incubated for 7–10 days and then supernatants were transferred to new 96 well plates. Then wells were stained with anti-GGV-NP antibody, which cross-reacts with the NPs of the viruses used in these experiments. Staining and washing was performed as previously described [23]. Stained plates were scanned on an Odyssey Licor instrument to identify infected wells. Supernatant from NP-positive wells were transferred to 24-well plate wells plated the day prior with 75,000 JK cells per well. One day later cell culture supernatant was replaced. After an additional 3 days of incubation, culture supernatant was collected and clarified by centrifugation at 10,000g for 1 minute. RNA was isolated from these supernatants using the ZR Viral RNA kit according to the manufacturer’s protocol (Zymo Research). RNA was used as template in qRT-PCR as described above to measure levels of viral RNA of various genotypes.

### Ethics statement

This study did not include experiments involving live animals. In some cases, samples (typically blood) were collected from live animals by attending veterinarians. In other cases, tissues were collected during necropsy. All samples were taken and used with owners consent. Some samples were collected in the context of other, related studies: The acquisition of tissue samples at the University of Florida was authorized under University of Florida Institutional Animal Care and Use Committee Protocol A116. The acquisition of samples at the University of California Davis was authorized under IACUC protocol 17450.

## Supporting Information

S1 Table Summary of snakes sequenced for this study. (XLSX)

S2 Table Sequences of oligonucleotides used in this study. (XLS)

S3 Table Counts and fractions of viral reads in datasets. (XLSX)

S1 Fig Cartoons depicting genome segment organization, coverage levels, and predicted secondary structure. Genome cartoons and features are drawn to scale. Vertical lines at the end of genome segments indicate that the putative terminal sequences are included in the assembly for that segment. Where applicable, partial coding sequences and the approximate location of recombination junctions are indicated. Note that it was not possible to confidently identify the recombination breakpoint for the L19 genome segments so it is not depicted. Below each cartoon are plotted coverage levels (the number of sequencing reads supporting each base in the assembly) and predicted free energy of folding (i.e. predicted RNA secondary structure; -∆G) of 140 nt sliding windows. **(A)** Cartoons and plots for all L segments. **(B)** Cartoons and plots for all S segments.
(PDF)

S2 Fig Relatedness of virus sequences within and between genotypes. **(A)** A histogram of L segment pairwise nucleotide identities. All pairs of L segments sequences were aligned and the global nucleotide identity calculated. Inter- and intra- genotype comparisons are colored as indicated. **(B)** Histogram for S segment sequences.
(PDF)

S3 Fig Phylogeny of representative snake and mammalian arenavirus S segments. Representative snake and mammalian arenavirus sequences were collected and used to create a multiple sequence alignment of NP CDS, which was used to create a Bayesian phylogeny. Red lines indicate Old World mammalian arenaviruses and blue lines New World viruses.
(PDF)

S4 Fig There are on average more than twice as many L segments as S segments in multiply infected animals. A histogram of the number of S and L genotypes detected in individual animals.
(PDF)

S5 Fig Possible mechanism of generation of 2xIGR recombinants. A cartoon depicting a possible mechanism for the generation of genome segments with two intergenic regions and partial coding sequences. During replication of the genome segment (1), the replication complex could disassociate from the original template (2), reassociate with another template in the cell (3), and replication could complete on the second template (4).
(PDF)

S6 Fig Discriminating qPCR corroborates sequencing data. Displayed are fractional abundances of indicated S or L segment genotypes in individual samples as measured by qRT-PCR using a panel of genotype-discriminating primers (q) and sequencing (s). Fractional abundance was measured for qPCR using standard curve-based quantitation and for sequencing using read mapping as in Fig 4. Neg snake is a sample from an uninfected snake and HeLa is total HeLa cell RNA. Asterisks (*) indicate the following issues related to template/primer compatibility: snake #30 L2 contains mismatches in the primer binding regions so doesn’t amplify; primers targeting the L3 genotype also amplify recombinant genotype L4 in snake #46 and #47; primers targeting the L18 genotype also amplify recombinant genotype L22 in snake #30. In these latter two cases, qPCR-measured abundance was split evenly between the two amplified genotypes.
(PDF)

S7 Fig Intrahost variation of individual genotypes. Polymorphic sites (minor alleles) were identified in sequencing data as described in Materials and Methods. The number of such variant sites was tallied in each genome segment and normalized to the length of the segment. A histogram of this normalized number for L and S segments is displayed in (A) and (B).
(PDF)
