# Supplementary material for: Widespread Recombination, Reassortment, and Transmission of Unbalanced Compound Viral Genotypes in Natural Arenavirus Infections
Source: PLoS Pathog. 2015 May 20;11(5):e1004900. doi: 10.1371/journal.ppat.1004900 (PMC4438980; doi:10.1371/journal.ppat.1004900)
Supplement: S1 Fig — Genome cartoons and features are drawn to scale. Vertical lines at the end of genome segments indicate that the putative terminal sequences are included in the assembly for that segment. Where applicable, partial coding sequences and the approximate location of recombination junctions are indicated. Note that it was not possible to confidently identify the recombination breakpoint for the L19 genome segments so it is not depicted. Below each cartoon are plotted coverage levels (the number of sequencing reads supporting each base in the assembly) and predicted free energy of folding (i.e. predicted RNA secondary structure; -∆G) of 140 nt sliding windows. (A) Cartoons and plots for all L segments. (B) Cartoons and plots for all S segments. (PDF) [file ppat.1004900.s004.pdf]

Supplemental Figure 1A

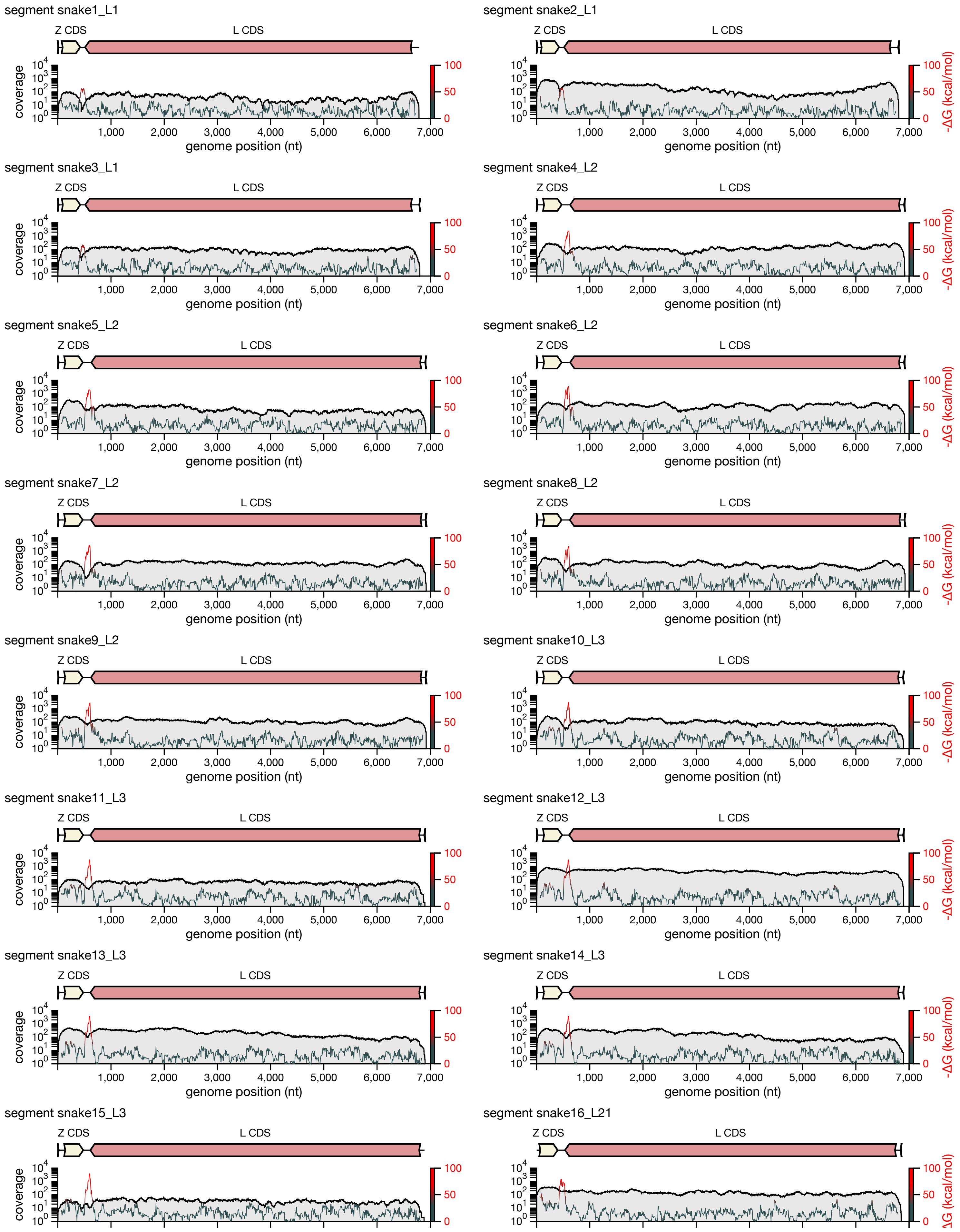

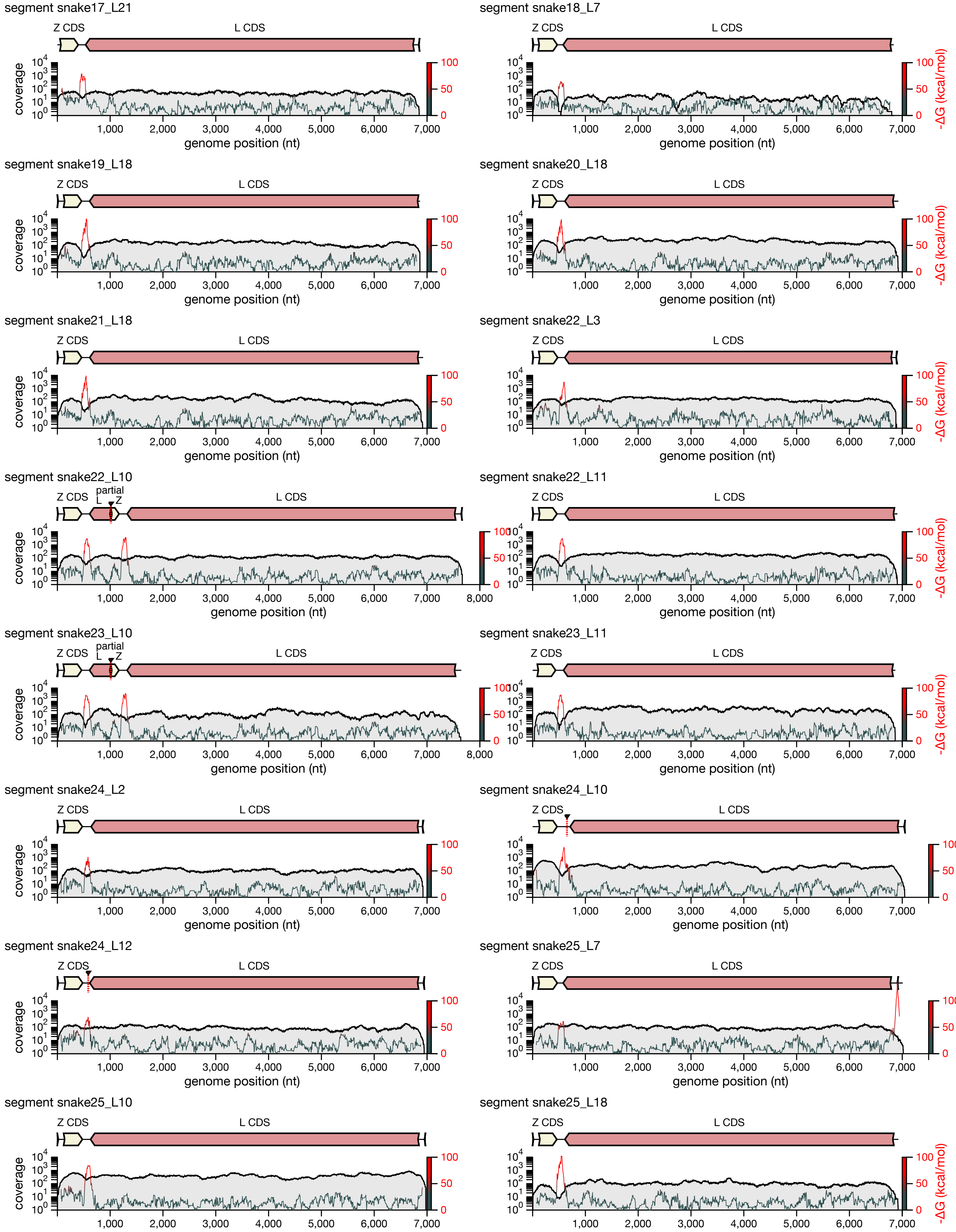

segment snake17_L21
segment snake18_L7
segment snake19_L18
segment snake20_L18
segment snake21_L18
segment snake22_L3
segment snake22_L10
segment snake22_L11
segment snake23_L10
segment snake23_L11
segment snake24_L2
segment snake24_L10
segment snake24_L12
segment snake25_L7
segment snake25_L10
segment snake25_L18
Z CDS
L CDS
partial
L
Z
coverage
10^0
10^1
10^2
10^3
10^4
0
50
100
-ΔG (kcal/mol)
1,000
2,000
3,000
4,000
5,000
6,000
7,000
8,000
genome position (nt)

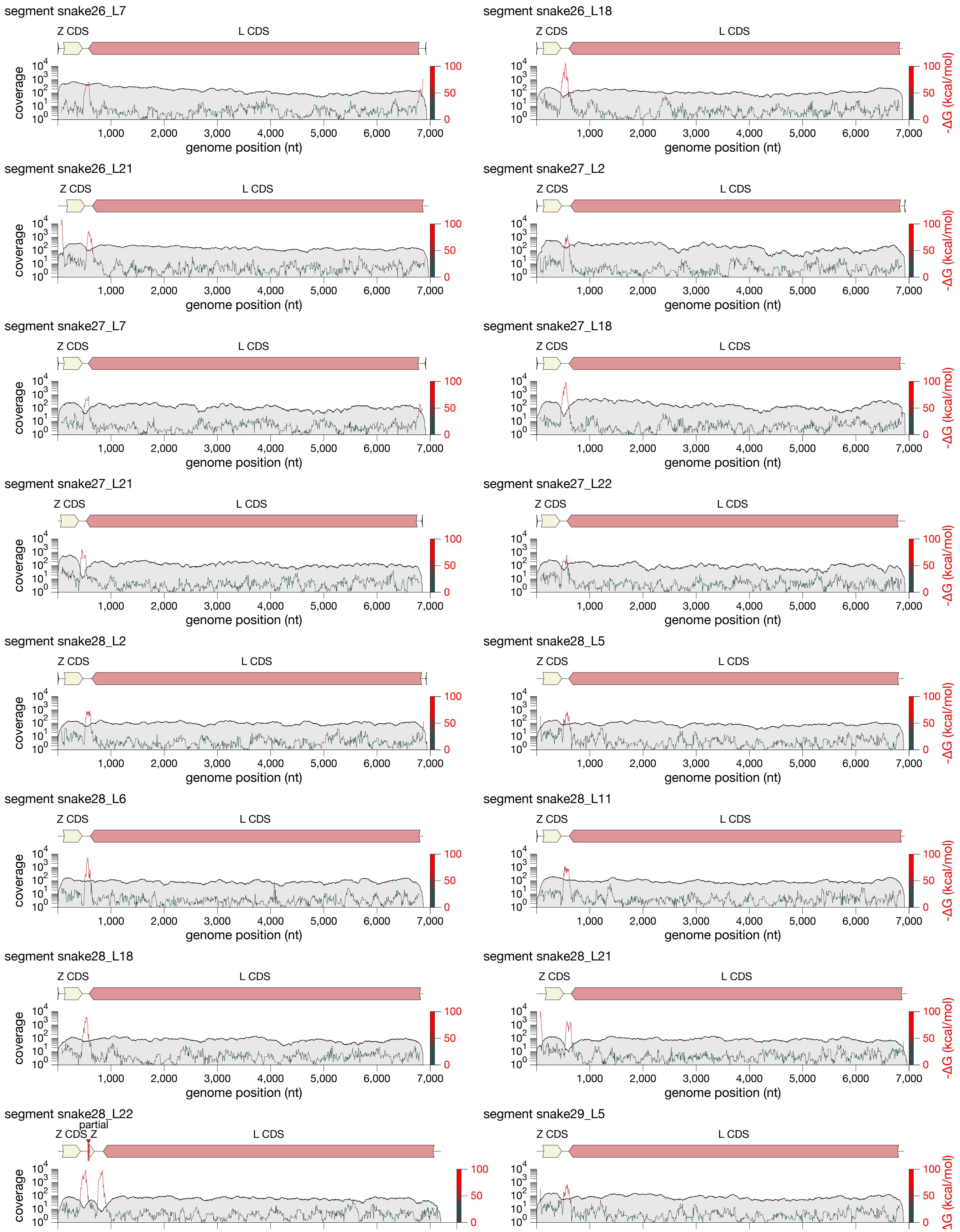

segment snake26_L7
Z CDS
L CDS
segment snake26_L18
Z CDS
L CDS
segment snake26_L21
Z CDS
L CDS
segment snake27_L2
Z CDS
L CDS
segment snake27_L7
Z CDS
L CDS
segment snake27_L18
Z CDS
L CDS
segment snake27_L21
Z CDS
L CDS
segment snake27_L22
Z CDS
L CDS
segment snake28_L2
Z CDS
L CDS
segment snake28_L5
Z CDS
L CDS
segment snake28_L6
Z CDS
L CDS
segment snake28_L11
Z CDS
L CDS
segment snake28_L18
Z CDS
L CDS
segment snake28_L21
Z CDS
L CDS
segment snake28_L22
partial
Z CDS Z
L CDS
segment snake29_L5
Z CDS
L CDS
coverage
genome position (nt)
-ΔG (kcal/mol)
1,000
2,000
3,000
4,000
5,000
6,000
7,000
100
50
0

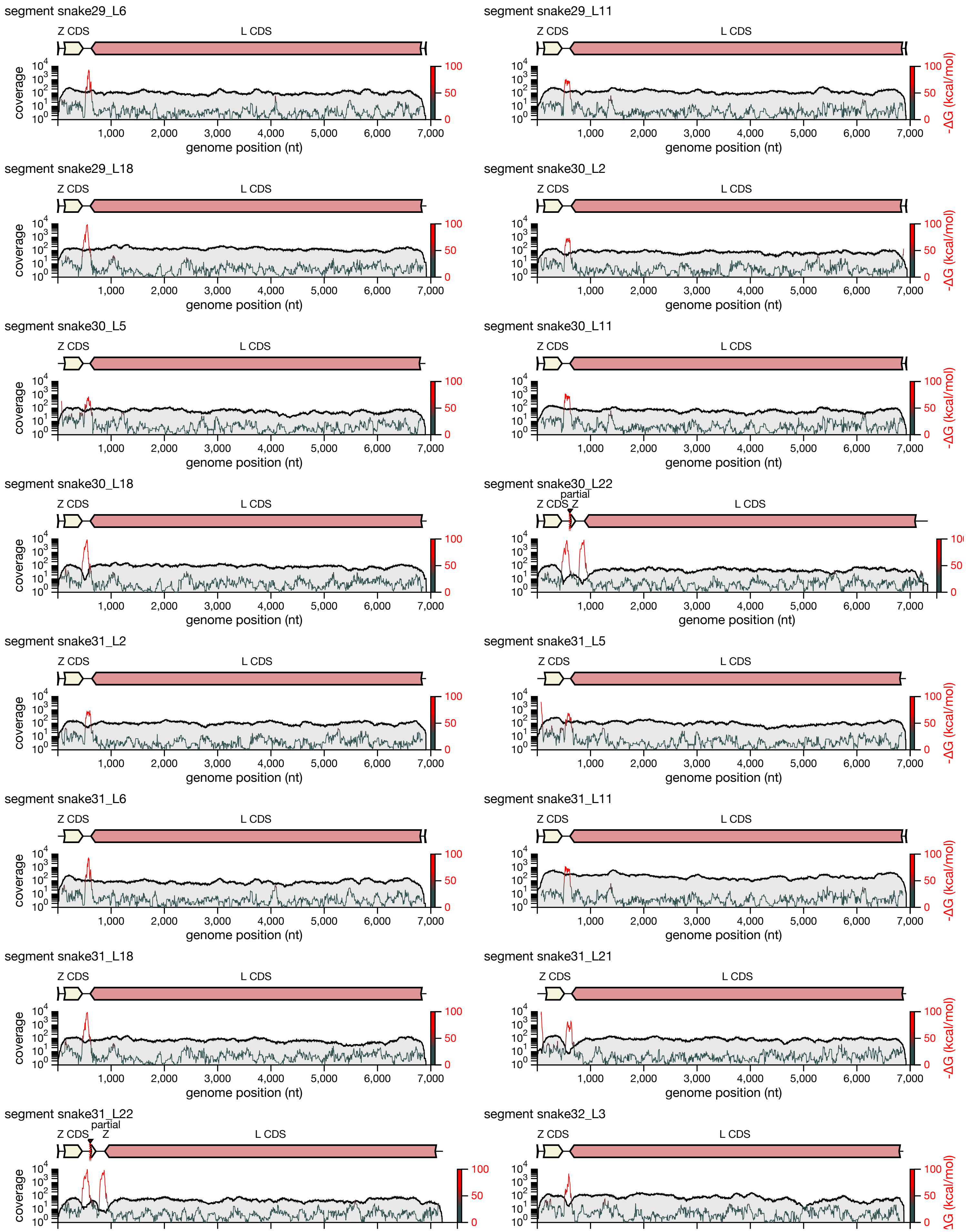

segment snake29_L6
segment snake29_L11
segment snake29_L18
segment snake30_L2
segment snake30_L5
segment snake30_L11
segment snake30_L18
segment snake30_L22
segment snake31_L2
segment snake31_L5
segment snake31_L6
segment snake31_L11
segment snake31_L18
segment snake31_L21
segment snake31_L22
segment snake32_L3
Z CDS
L CDS
partial Z
coverage
genome position (nt)
-ΔG (kcal/mol)

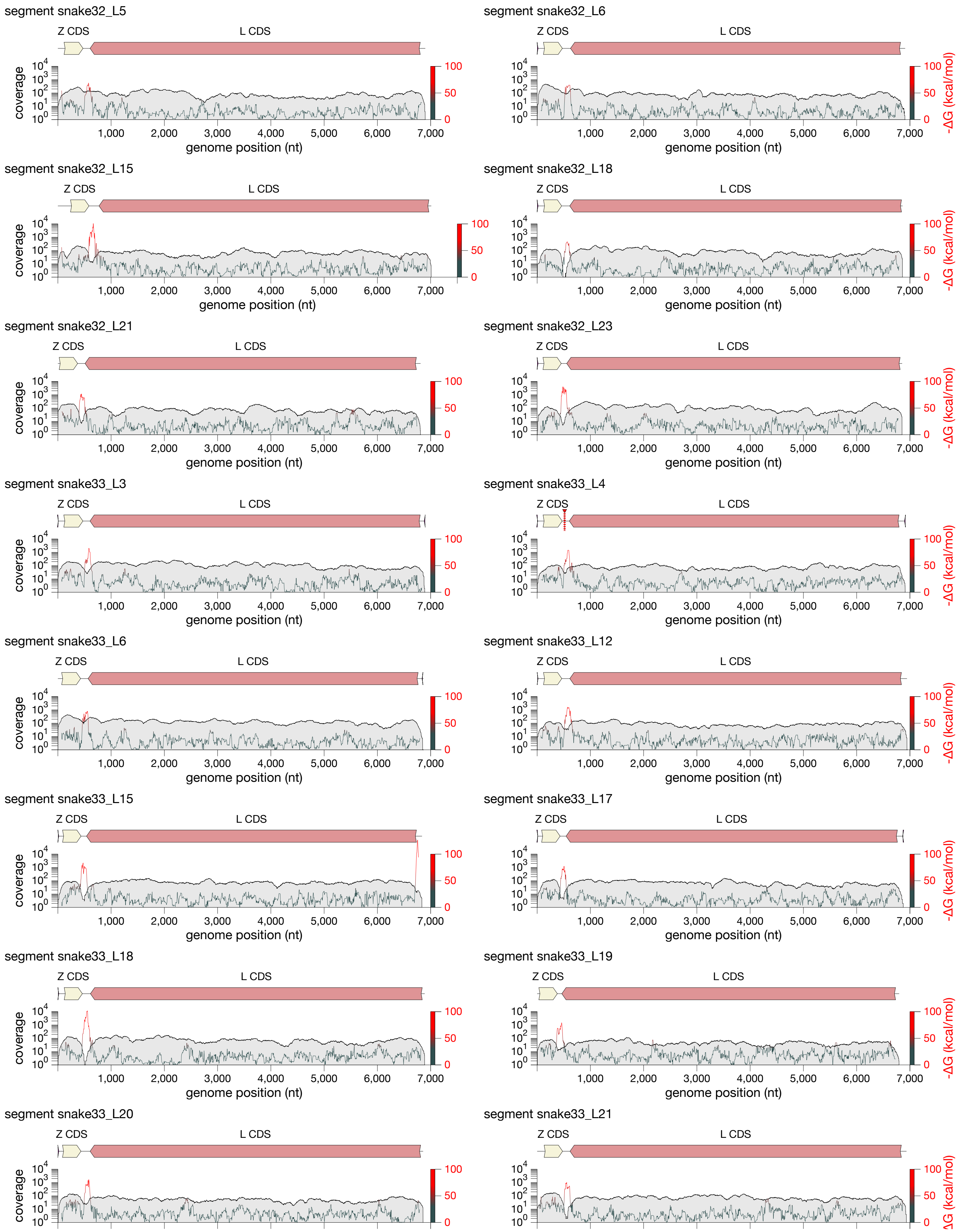
segment snake32_L5
segment snake32_L6
segment snake32_L15
segment snake32_L18
segment snake32_L21
segment snake32_L23
segment snake33_L3
segment snake33_L4
segment snake33_L6
segment snake33_L12
segment snake33_L15
segment snake33_L17
segment snake33_L18
segment snake33_L19
segment snake33_L20
segment snake33_L21
Z CDS
L CDS
coverage
10^0
10^1
10^2
10^3
10^4
1,000
2,000
3,000
4,000
5,000
6,000
7,000
genome position (nt)
0
50
100
-ΔG (kcal/mol)

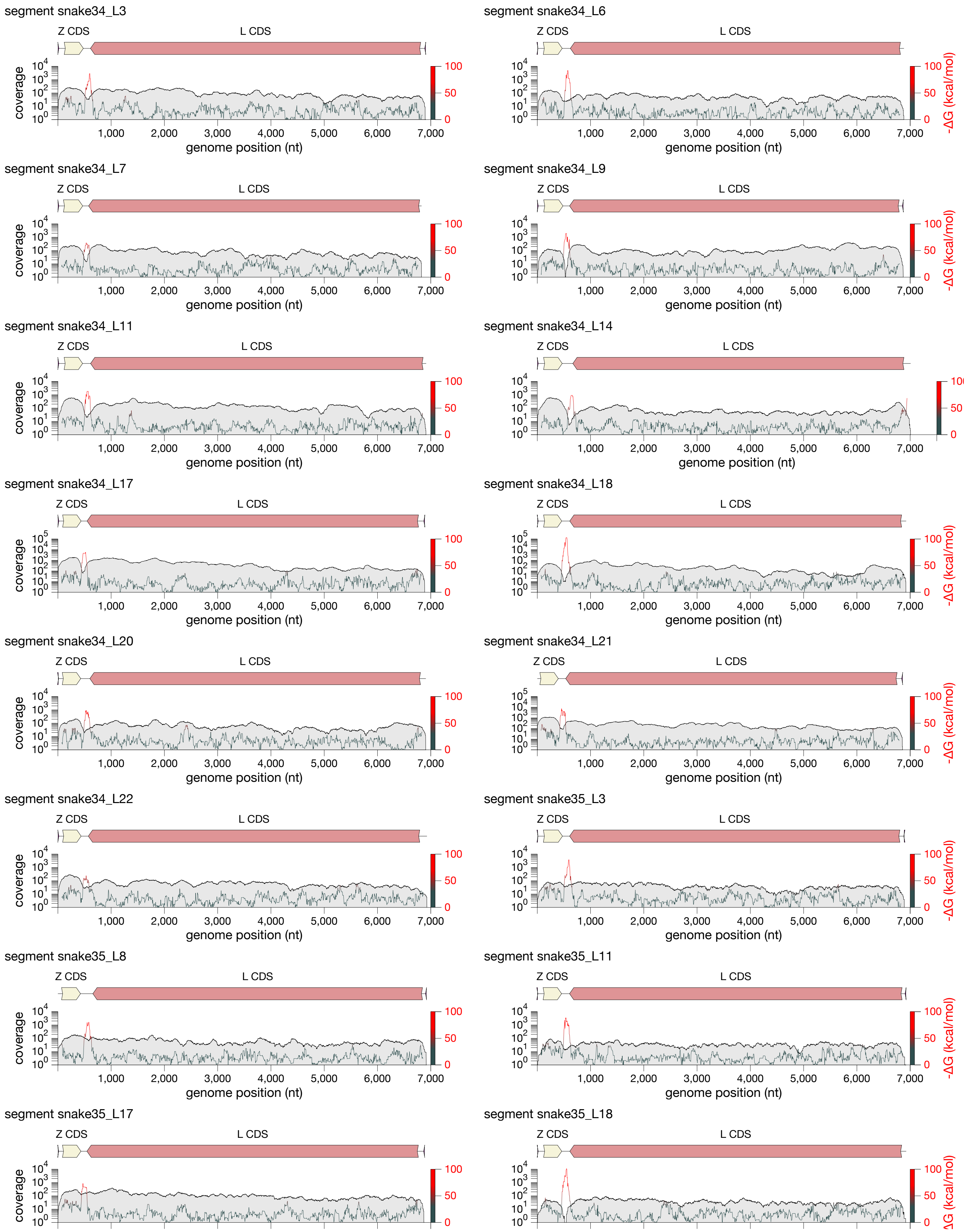
segment snake34_L3
segment snake34_L6
segment snake34_L7
segment snake34_L9
segment snake34_L11
segment snake34_L14
segment snake34_L17
segment snake34_L18
segment snake34_L20
segment snake34_L21
segment snake34_L22
segment snake35_L3
segment snake35_L8
segment snake35_L11
segment snake35_L17
segment snake35_L18
Z CDS
L CDS
coverage
genome position (nt)
-ΔG (kcal/mol)
1,000
2,000
3,000
4,000
5,000
6,000
7,000
0
50
100

segment snake35_L21

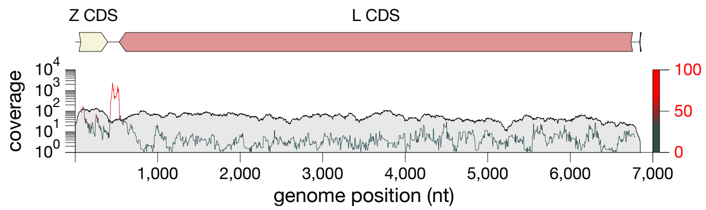

segment snake36_L3

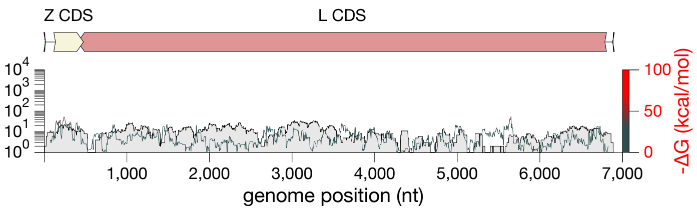

segment snake36_L11

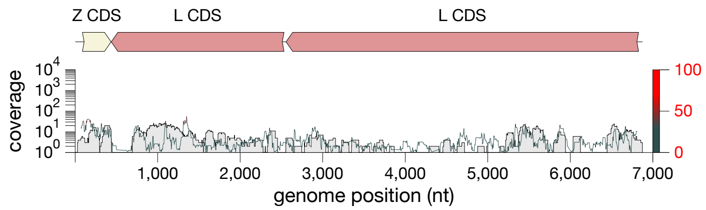

segment snake36_L17

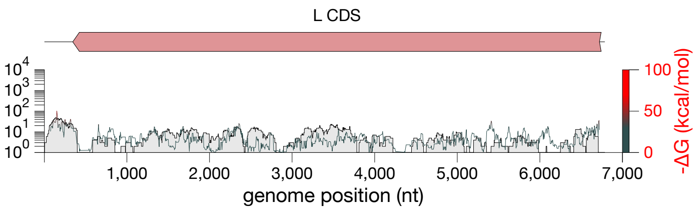

segment snake36_L21

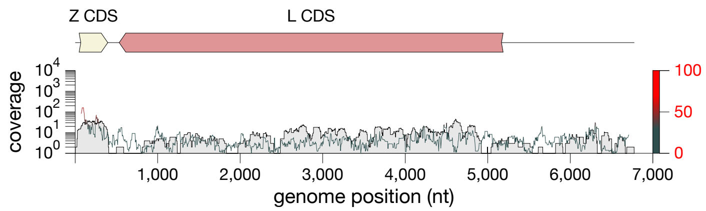

segment snake37_L3

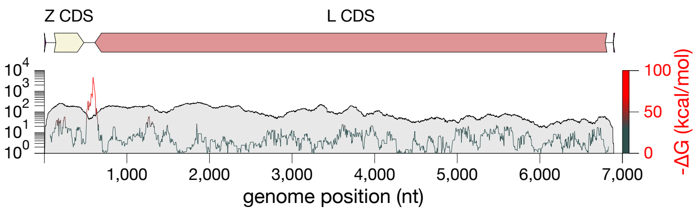

segment snake37_L13

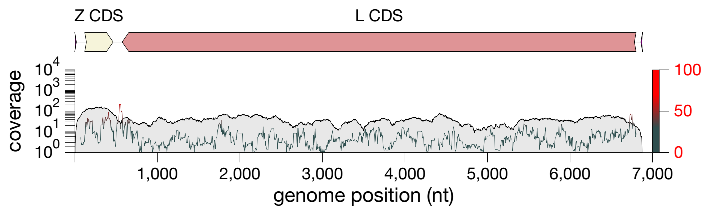

segment snake37_L21

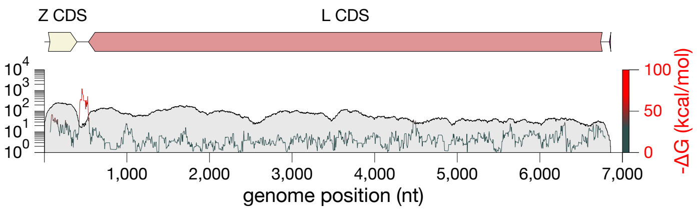

segment snake38_L3

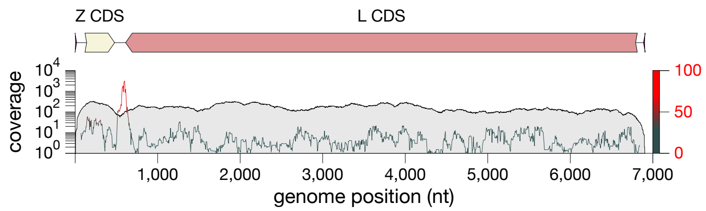

segment snake38_L18

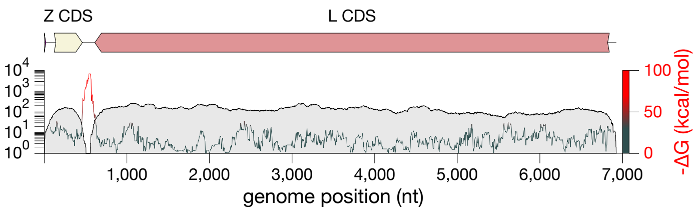

segment snake39_L3

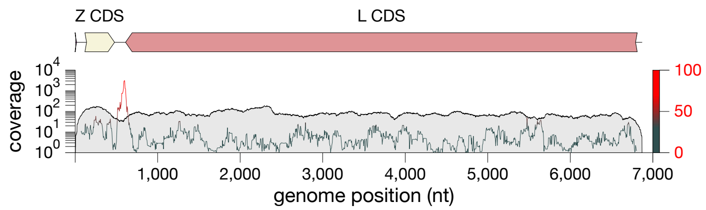

segment snake39_L18

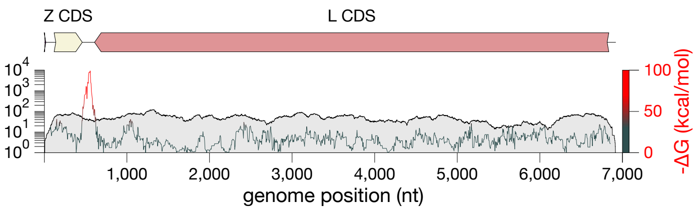

segment snake40_L3

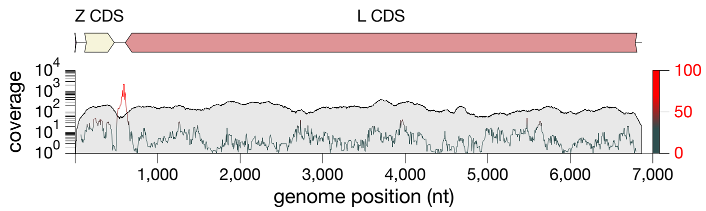

segment snake40_L6

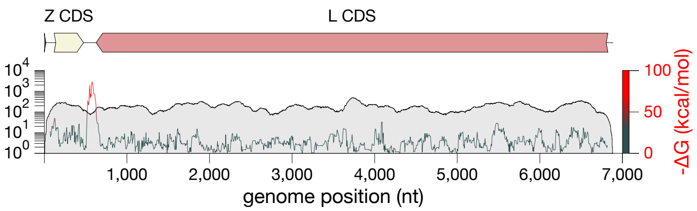

segment snake40_L7

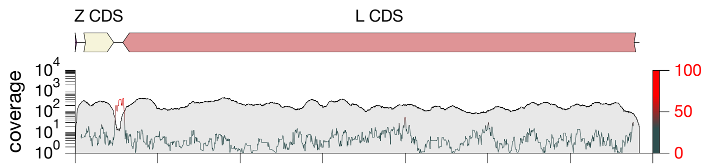

segment snake40_L8

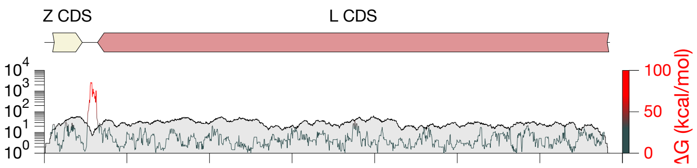

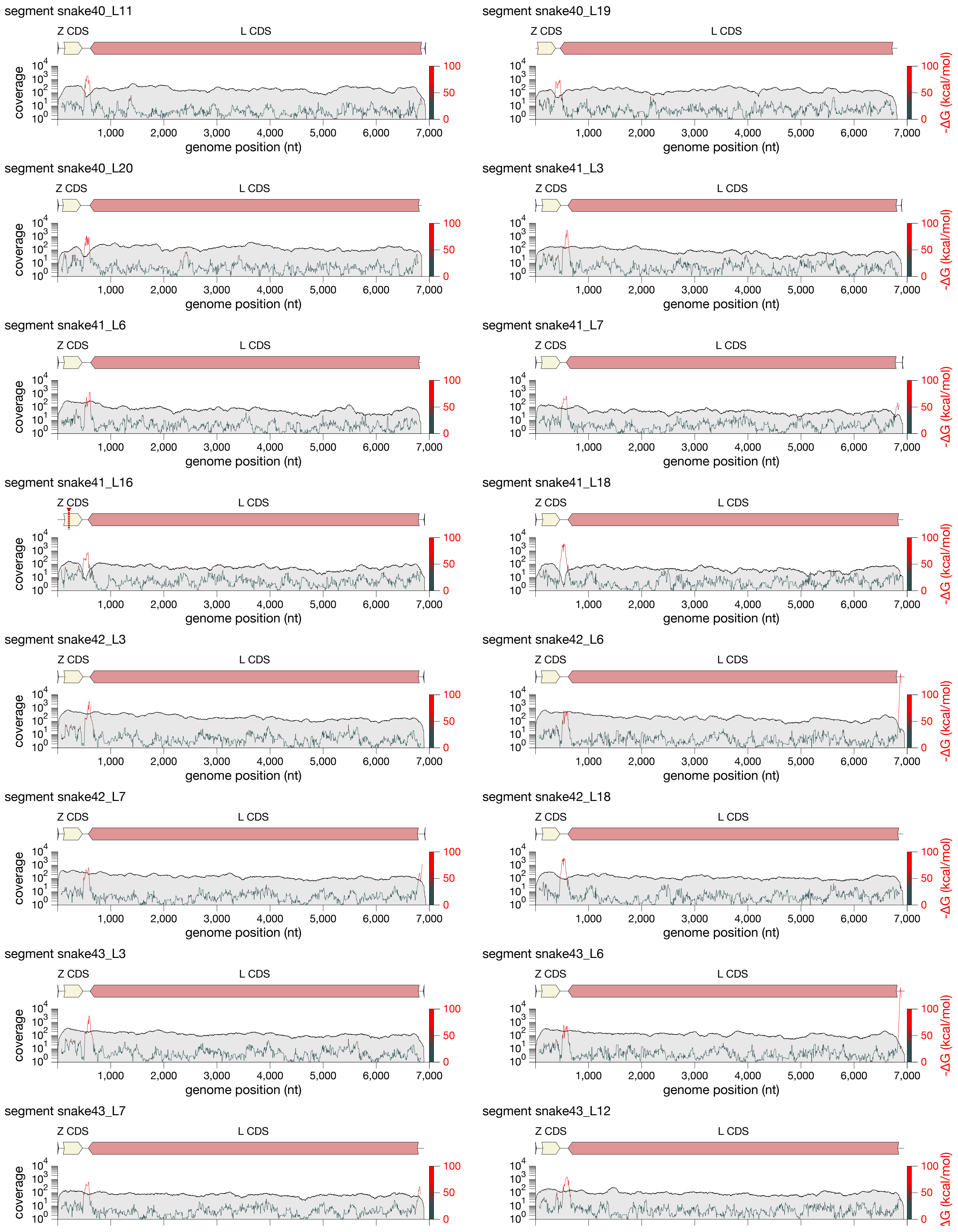

segment snake40_L11
segment snake40_L19
segment snake40_L20
segment snake41_L3
segment snake41_L6
segment snake41_L7
segment snake41_L16
segment snake41_L18
segment snake42_L3
segment snake42_L6
segment snake42_L7
segment snake42_L18
segment snake43_L3
segment snake43_L6
segment snake43_L7
segment snake43_L12
Z CDS
L CDS
coverage
genome position (nt)
-ΔG (kcal/mol)

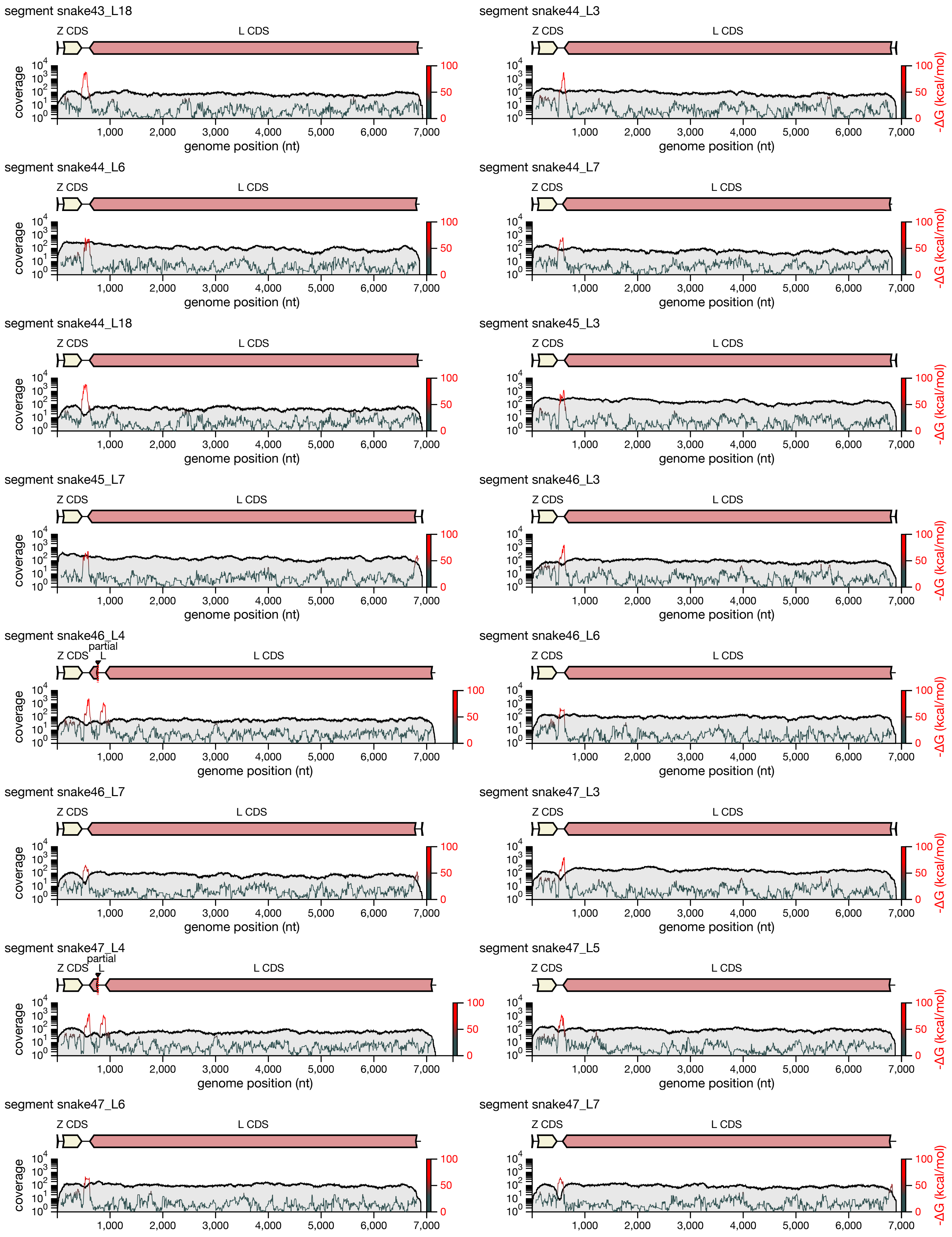

segment snake43_L18
segment snake44_L3
segment snake44_L6
segment snake44_L7
segment snake44_L18
segment snake45_L3
segment snake45_L7
segment snake46_L3
segment snake46_L4
segment snake46_L6
segment snake46_L7
segment snake47_L3
segment snake47_L4
segment snake47_L5
segment snake47_L6
segment snake47_L7
Z CDS
L CDS
partial L
coverage
genome position (nt)
-ΔG (kcal/mol)
10^0
10^1
10^2
10^3
10^4
0
50
100
1,000
2,000
3,000
4,000
5,000
6,000
7,000

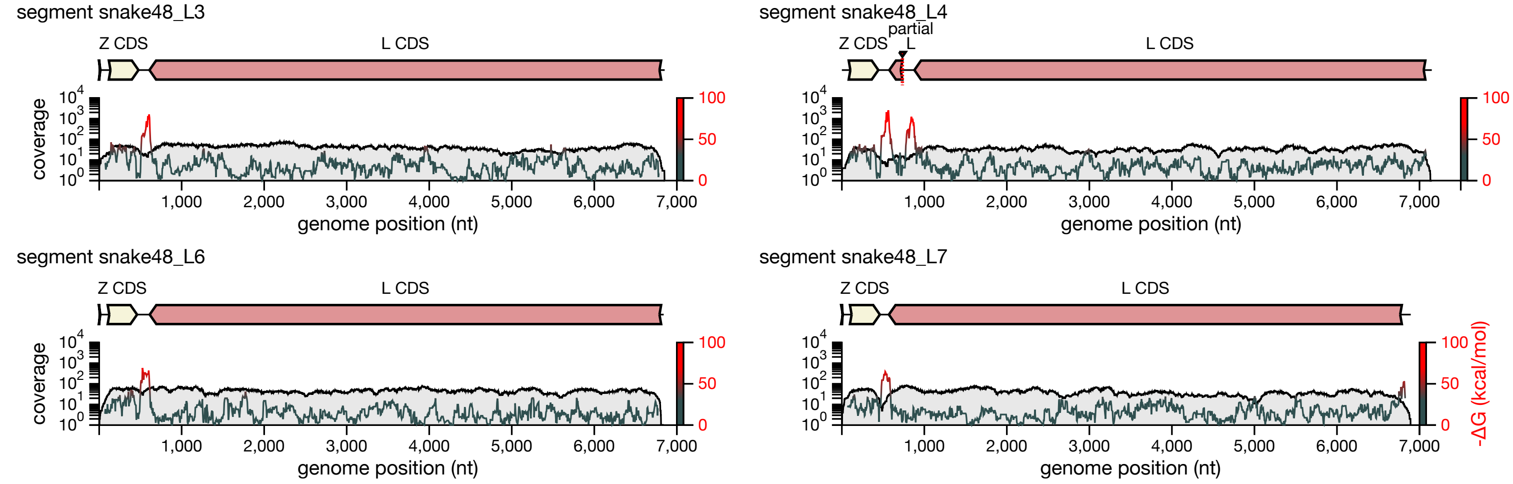

S1 Fig: Cartoons depicting genome segment organization, coverage levels, and predicted secondary structure. Genome cartoons and features are drawn to scale. Vertical lines at the end of genome segments indicate that the putative terminal sequences are included in the assembly for that segment. Where applicable, partial coding sequences and the approximate location of recombination junctions are indicated. Note that it was not possible to confidently identify the recombination breakpoint for the L19 genome segments so it is not depicted. Below each cartoon are plotted coverage levels (the number of sequencing reads supporting each base in the assembly) and predicted free energy of folding (i.e. predicted RNA secondary structure; -ΔG) of 140 nt sliding windows. **(A)** Cartoons and plots for all L segments. **(B)** Cartoons and plots for all S segments.

Supplemental Figure 1B

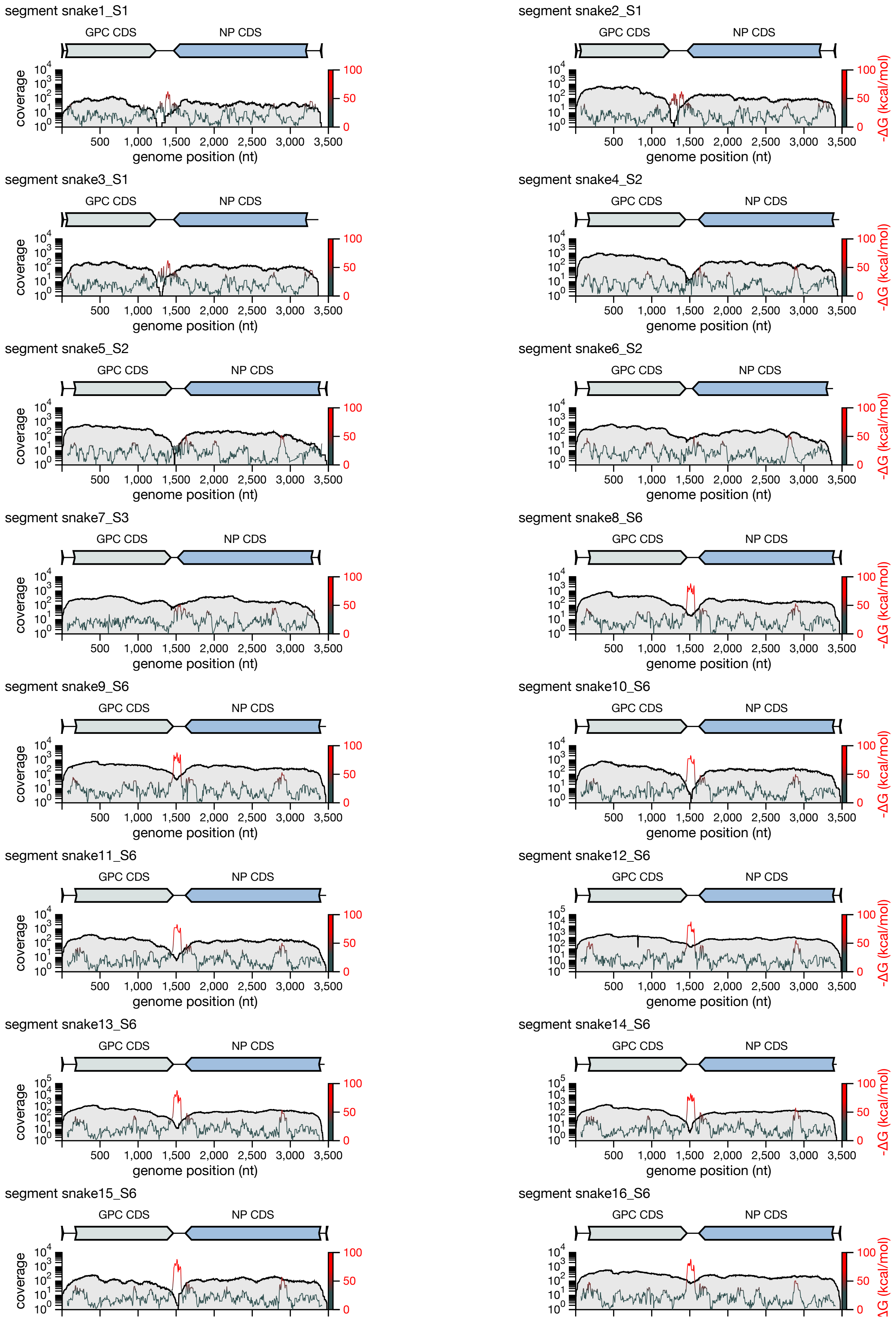

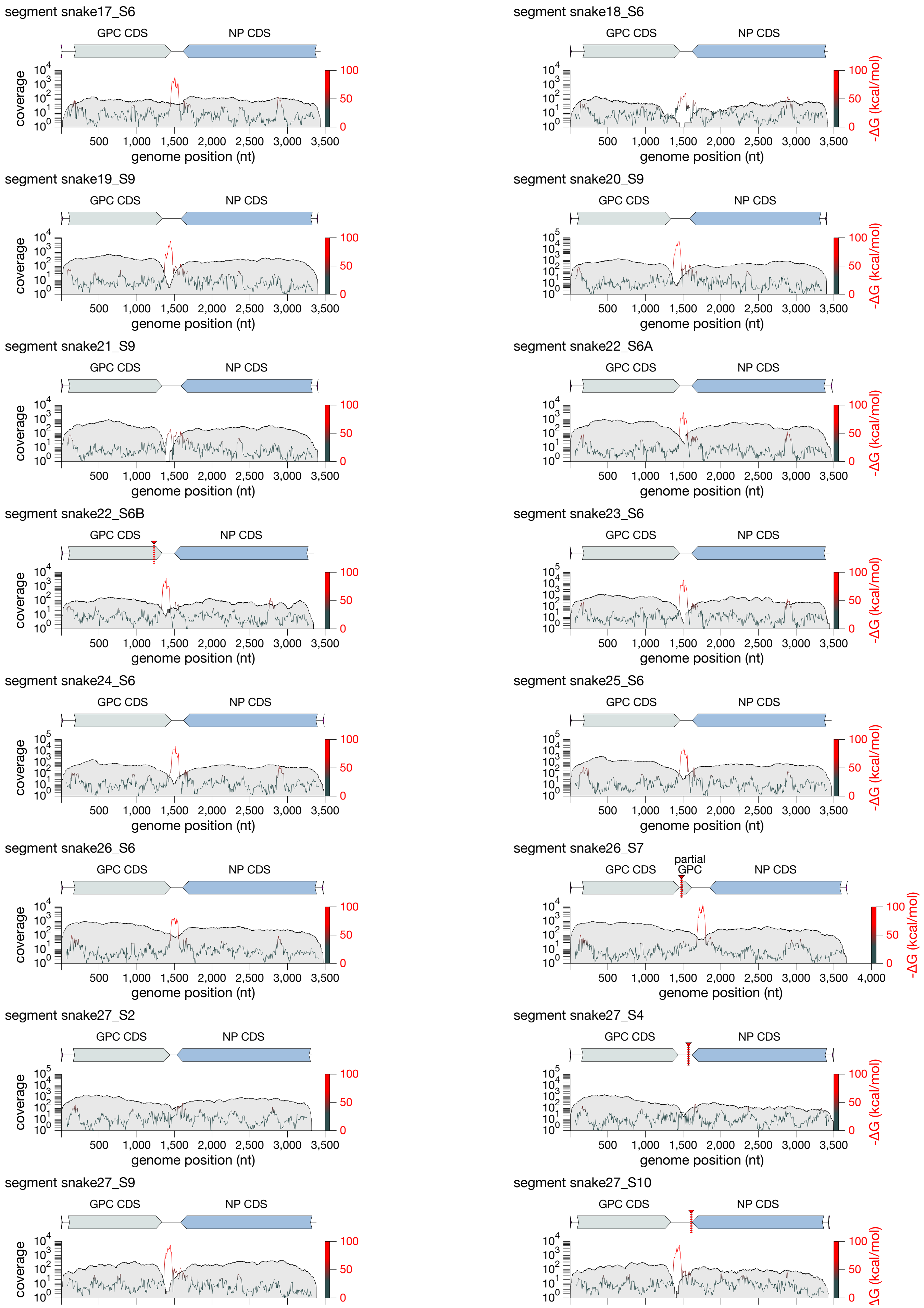
segment snake17_S6
GPC CDS
NP CDS
coverage
genome position (nt)
segment snake18_S6
GPC CDS
NP CDS
coverage
genome position (nt)
-ΔG (kcal/mol)
segment snake19_S9
GPC CDS
NP CDS
coverage
genome position (nt)
segment snake20_S9
GPC CDS
NP CDS
coverage
genome position (nt)
-ΔG (kcal/mol)
segment snake21_S9
GPC CDS
NP CDS
coverage
genome position (nt)
segment snake22_S6A
GPC CDS
NP CDS
coverage
genome position (nt)
-ΔG (kcal/mol)
segment snake22_S6B
GPC CDS
NP CDS
coverage
genome position (nt)
segment snake23_S6
GPC CDS
NP CDS
coverage
genome position (nt)
-ΔG (kcal/mol)
segment snake24_S6
GPC CDS
NP CDS
coverage
genome position (nt)
segment snake25_S6
GPC CDS
NP CDS
coverage
genome position (nt)
-ΔG (kcal/mol)
segment snake26_S6
GPC CDS
NP CDS
coverage
genome position (nt)
segment snake26_S7
GPC CDS
partial GPC
NP CDS
coverage
genome position (nt)
-ΔG (kcal/mol)
segment snake27_S2
GPC CDS
NP CDS
coverage
genome position (nt)
segment snake27_S4
GPC CDS
NP CDS
coverage
genome position (nt)
-ΔG (kcal/mol)
segment snake27_S9
GPC CDS
NP CDS
coverage
segment snake27_S10
GPC CDS
NP CDS
coverage

segment snake28_S6

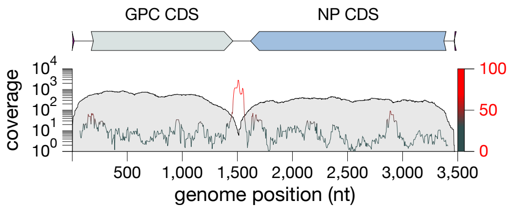

segment snake29_S6

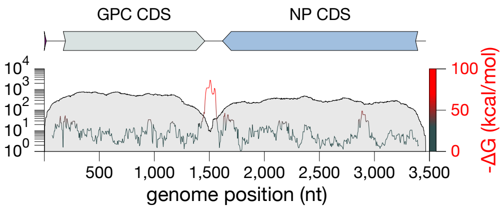

segment snake30_S6

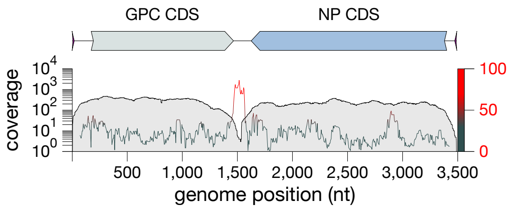

segment snake30_S9

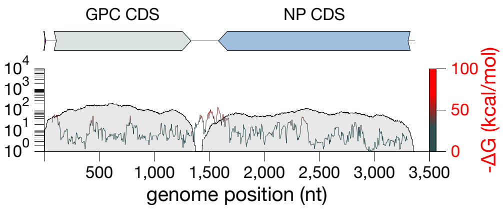

segment snake31_S6

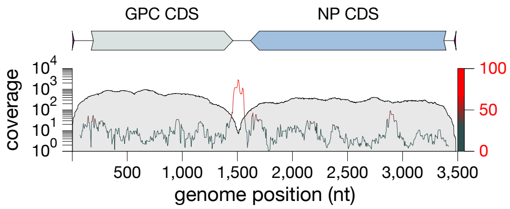

segment snake32_S6

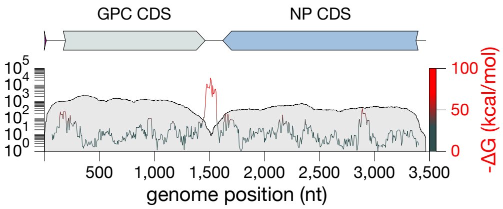

segment snake33_S6

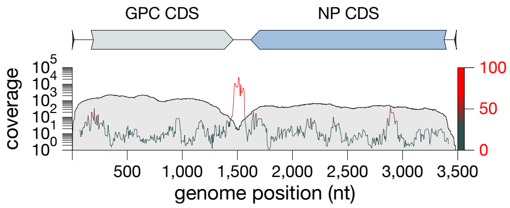

segment snake34_S6

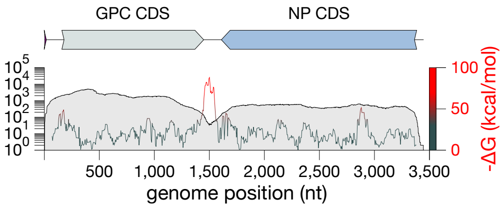

segment snake34_S9

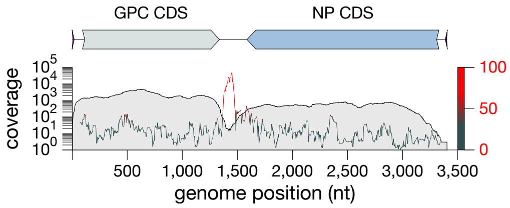

segment snake34_S10

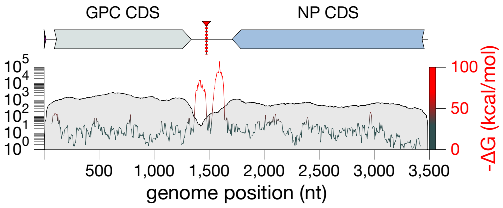

segment snake34_S11

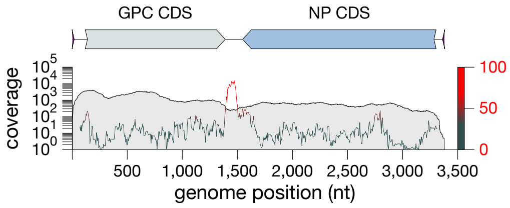

segment snake35_S6

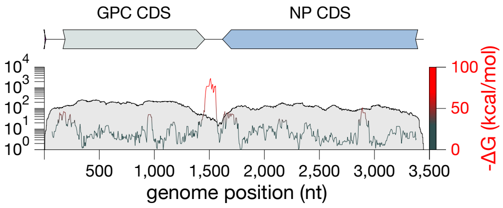

segment snake35_S8

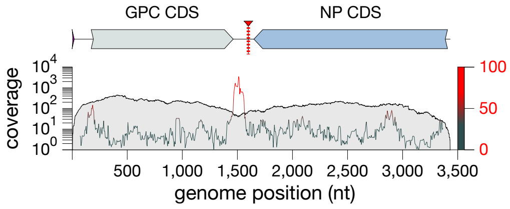

segment snake36_S6

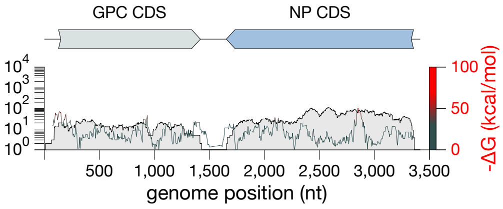

segment snake37_S6

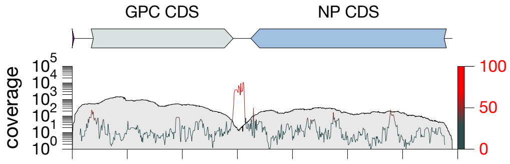

segment snake38_S6

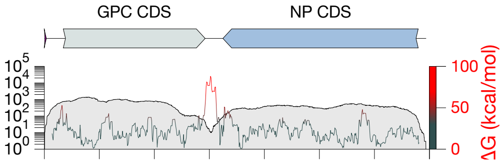

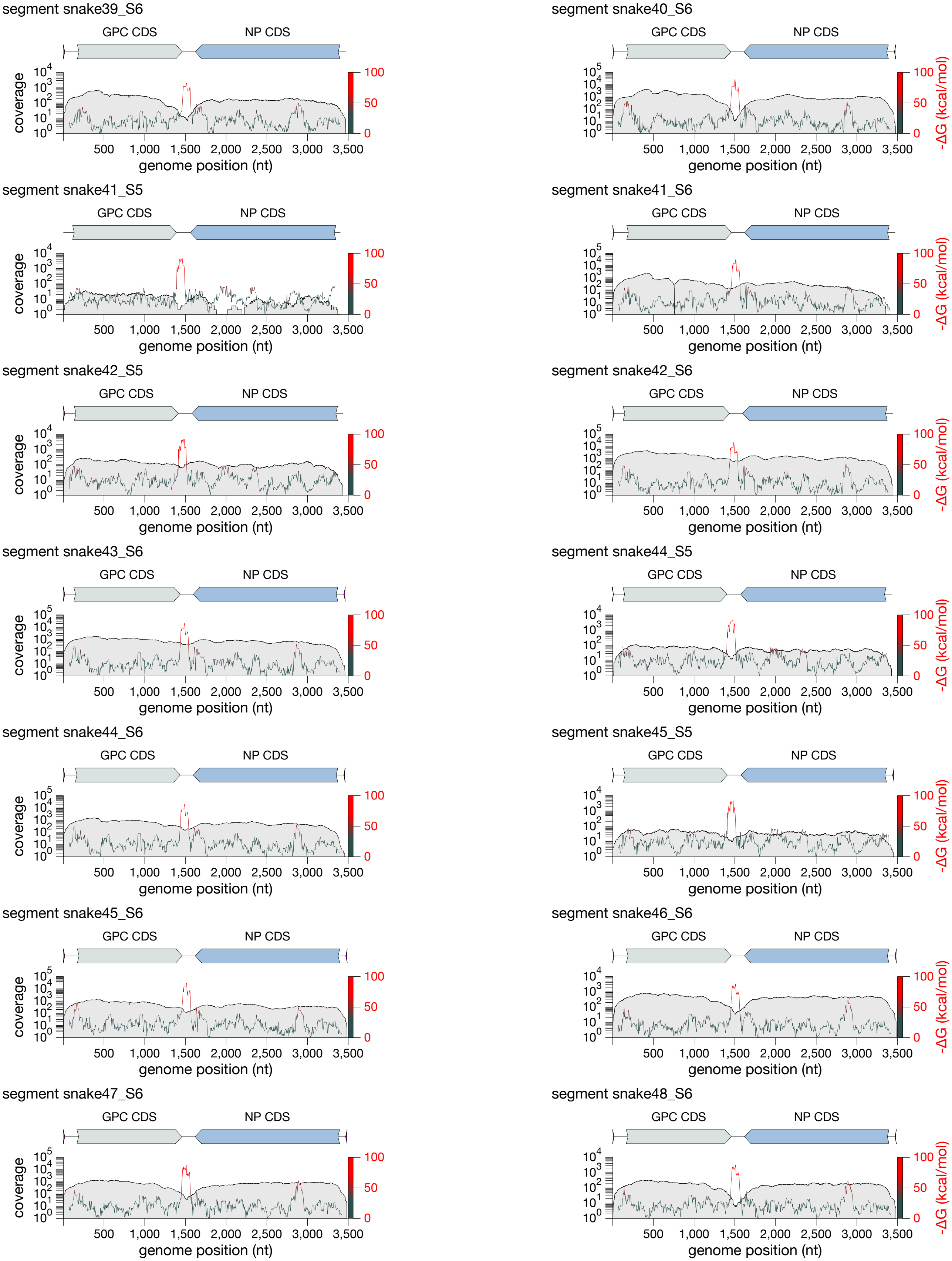
segment snake39_S6
segment snake40_S6
segment snake41_S5
segment snake41_S6
segment snake42_S5
segment snake42_S6
segment snake43_S6
segment snake44_S5
segment snake44_S6
segment snake45_S5
segment snake45_S6
segment snake46_S6
segment snake47_S6
segment snake48_S6
GPC CDS
NP CDS
coverage
genome position (nt)
-ΔG (kcal/mol)
500
1,000
1,500
2,000
2,500
3,000
3,500
100
50
0
