# Supplementary material for: Widespread Recombination, Reassortment, and Transmission of Unbalanced Compound Viral Genotypes in Natural Arenavirus Infections
Source: PLoS Pathog. 2015 May 20;11(5):e1004900. doi: 10.1371/journal.ppat.1004900 (PMC4438980; doi:10.1371/journal.ppat.1004900)
Supplement: S2 Fig — (A) A histogram of L segment pairwise nucleotide identities. All pairs of L segments sequences were aligned and the global nucleotide identity calculated. Inter- and intra- genotype comparisons are colored as indicated. (B) Histogram for S segment sequences. (PDF) [file ppat.1004900.s005.pdf]

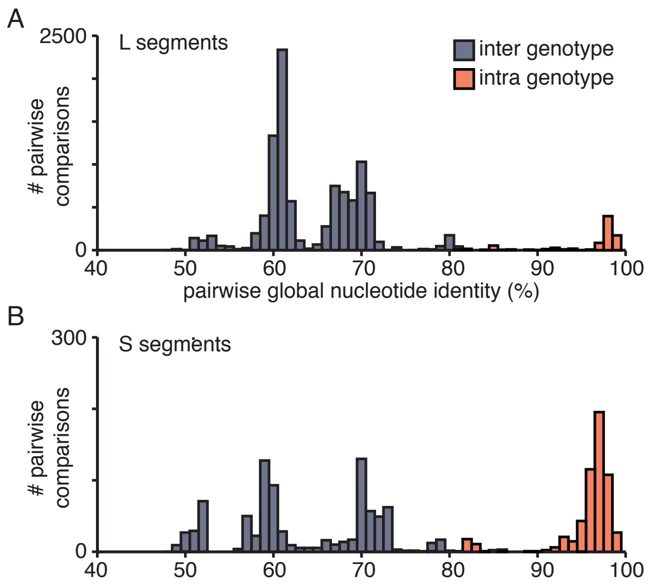

S2 Fig: Relatedness of virus sequences within and between genotypes. **(A)** A histogram of L segment pairwise nucleotide identities. All pairs of L segments sequences were aligned and the global nucleotide identity calculated. Inter- and intra-genotype comparisons are colored as indicated. **(B)** Histogram for S segment sequences.
