# Supplementary material for: Widespread Recombination, Reassortment, and Transmission of Unbalanced Compound Viral Genotypes in Natural Arenavirus Infections
Source: PLoS Pathog. 2015 May 20;11(5):e1004900. doi: 10.1371/journal.ppat.1004900 (PMC4438980; doi:10.1371/journal.ppat.1004900)
Supplement: S3 Fig — Representative snake and mammalian arenavirus sequences were collected and used to create a multiple sequence alignment of NP CDS, which was used to create a Bayesian phylogeny. Red lines indicate Old World mammalian arenaviruses and blue lines New World viruses. (PDF) [file ppat.1004900.s006.pdf]

NP CDS phylogeny

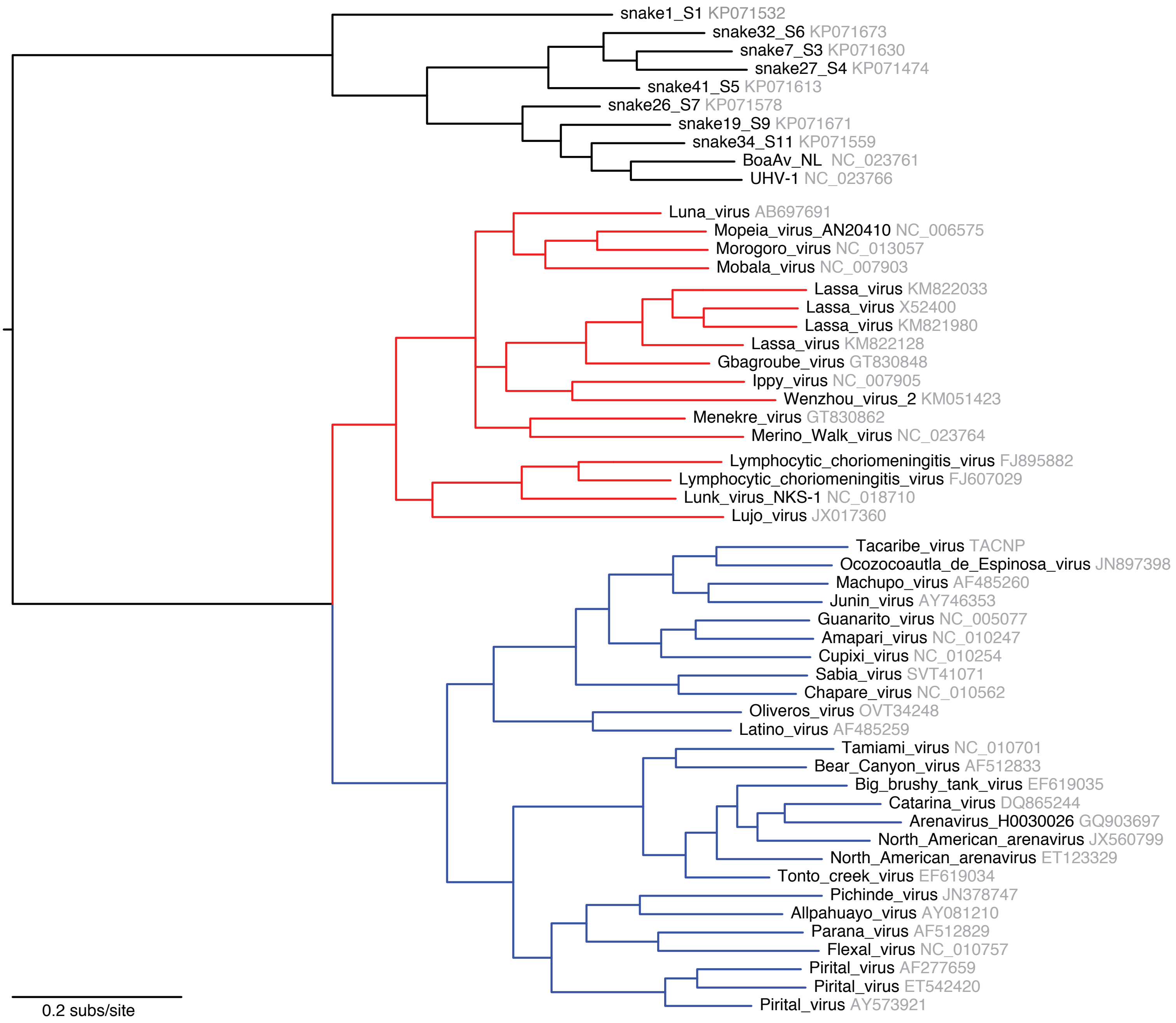

S3 Fig: **Phylogeny of representative snake and mammalian arenavirus S segments**. Representative snake and mammalian arenavirus sequences were collected and used to create a multiple sequence alignment of the NP CDS, which were used to create a Bayesian phylogeny. Red lines indicate Old World mammalian arenaviruses and blue lines New World viruses. Sequences accession numbers are indicated.
