# Supplementary material for: Widespread Recombination, Reassortment, and Transmission of Unbalanced Compound Viral Genotypes in Natural Arenavirus Infections
Source: PLoS Pathog. 2015 May 20;11(5):e1004900. doi: 10.1371/journal.ppat.1004900 (PMC4438980; doi:10.1371/journal.ppat.1004900)
Supplement: S4 Fig — A histogram of the number of S and L genotypes detected in individual animals. (PDF) [file ppat.1004900.s007.pdf]

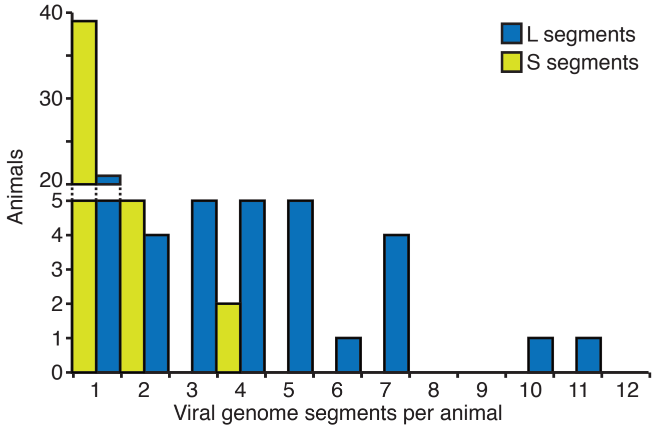

S4 Fig: There are on average more than twice as many L segments as S segments in multiply infected animals. A histogram of the number of S and L genotypes detected in individual animals.
