# Supplementary material for: Widespread Recombination, Reassortment, and Transmission of Unbalanced Compound Viral Genotypes in Natural Arenavirus Infections
Source: PLoS Pathog. 2015 May 20;11(5):e1004900. doi: 10.1371/journal.ppat.1004900 (PMC4438980; doi:10.1371/journal.ppat.1004900)
Supplement: S5 Fig — A cartoon depicting a possible mechanism for the generation of genome segments with two intergenic regions and partial coding sequences. During replication of the genome segment (1), the replication complex could disassociate from the original template (2), reassociate with another template in the cell (3), and replication could complete on the second template (4). (PDF) [file ppat.1004900.s008.pdf]

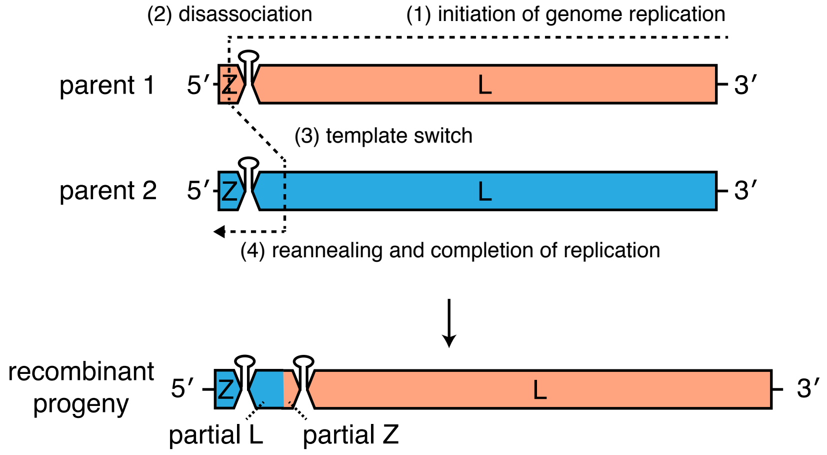

S5 Fig: Possible mechanism of generation of 2xIGR recombinants. A cartoon depicting a possible mechanism for the generation of genome segments with two intergenic regions and partial coding sequences. During replication of the genome segment (1), the replication complex could disassociate from the original template (2), reassociate with another template in the cell (3), and replication could complete on the second template (4).
