# Supplementary material for: Widespread Recombination, Reassortment, and Transmission of Unbalanced Compound Viral Genotypes in Natural Arenavirus Infections
Source: PLoS Pathog. 2015 May 20;11(5):e1004900. doi: 10.1371/journal.ppat.1004900 (PMC4438980; doi:10.1371/journal.ppat.1004900)
Supplement: S6 Fig — Displayed are fractional abundances of indicated S or L segment genotypes in individual samples as measured by qRT-PCR using a panel of genotype-discriminating primers (q) and sequencing (s). Fractional abundance was measured for qPCR using standard curve-based quantitation and for sequencing using read mapping as in Fig 4. Neg snake is a sample from an uninfected snake and HeLa is total HeLa cell RNA. Asterisks (*) indicate the following issues related to template/primer compatibility: snake #30 L2 contains mismatches in the primer binding regions so doesn’t amplify; primers targeting the L3 genotype also amplify recombinant genotype L4 in snake #46 and #47; primers targeting the L18 genotype also amplify recombinant genotype L22 in snake #30. In these latter two cases, qPCR-measured abundance was split evenly between the two amplified genotypes. (PDF) [file ppat.1004900.s009.pdf]

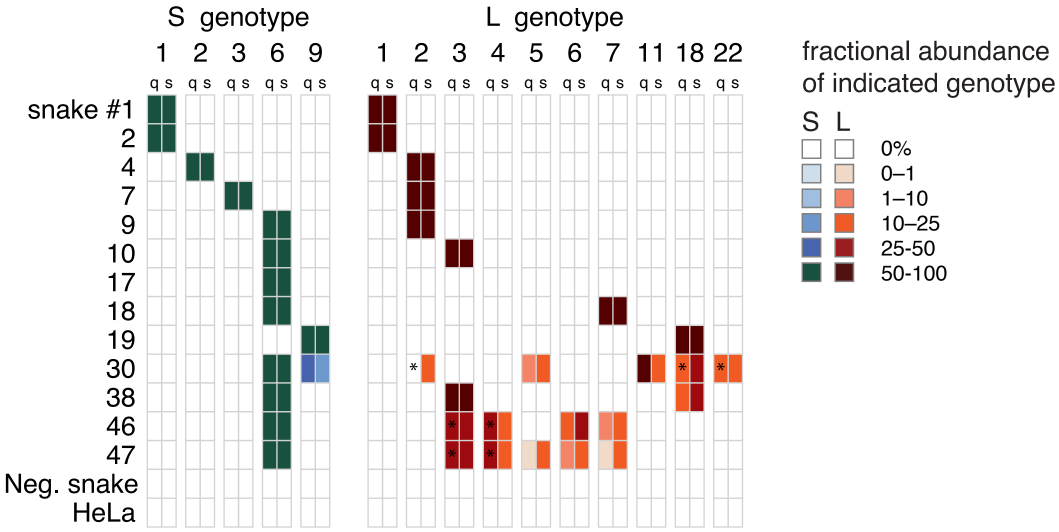

S6 Fig: Discriminating qPCR corroborates sequencing data. Displayed are fractional abundances of indicated S or L segment genotypes in individual samples as measured by qRT-PCR using a panel of genotype-discriminating primers (q) and sequencing (s). Fractional abundance was measured for qPCR using standard curve-based quantitation and for sequencing using read mapping as in Figure 2. Neg snake is a sample from an uninfected snake and HeLa is total HeLa cell RNA. Asterisks (*) indicate the following issues related to template/primer compatibility: snake #30 L2 contains mismatches in the primer binding regions so doesn't amplify; primers targeting the L3 genotype also amplify recombinant genotype L4 in snake #46 and #47; primers targeting the L18 genotype also amplify recombinant genotype L22 in snake #30. In these latter two cases, qPCR-measured abundance was split evenly between the two amplified genotypes.
