# Supplementary material for: Widespread Recombination, Reassortment, and Transmission of Unbalanced Compound Viral Genotypes in Natural Arenavirus Infections
Source: PLoS Pathog. 2015 May 20;11(5):e1004900. doi: 10.1371/journal.ppat.1004900 (PMC4438980; doi:10.1371/journal.ppat.1004900)
Supplement: S7 Fig — Polymorphic sites (minor alleles) were identified in sequencing data as described in Materials and Methods. The number of such variant sites was tallied in each genome segment and normalized to the length of the segment. A histogram of this normalized number for L and S segments is displayed in (A) and (B). (PDF) [file ppat.1004900.s010.pdf]

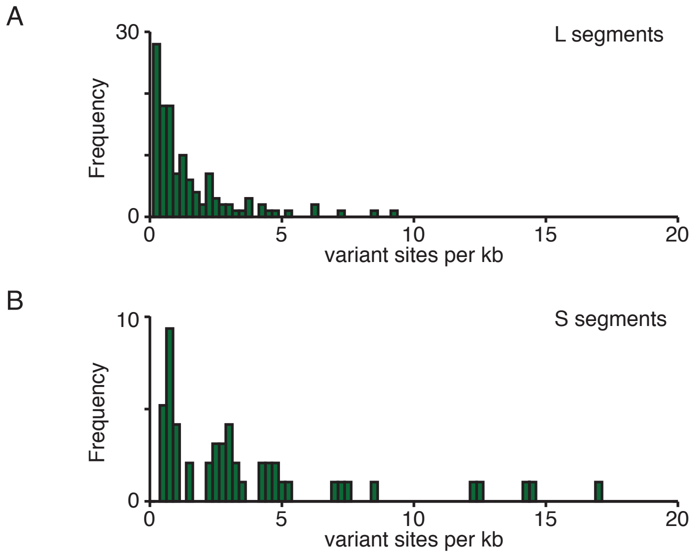

S7 Fig: Intrahost variation of individual genotypes. Polymorphic sites (minor alleles) were identified in sequencing data as described in Materials and Methods. The number of such variant sites was tallied in each genome segment and normalized to the length of the segment. A histogram of this normalized number for L and S segments is displayed in (A) and (B).
